# Differential Solvent DEEP-STD NMR and MD Simulations Enable the Determinants of the Molecular Recognition of Heparin Oligosaccharides by Antithrombin to Be Disentangled

**DOI:** 10.3390/ijms25094669

**Published:** 2024-04-25

**Authors:** Michela Parafioriti, Stefano Elli, Juan C. Muñoz-García, Jonathan Ramírez-Cárdenas, Edwin A. Yates, Jesús Angulo, Marco Guerrini

**Affiliations:** 1 Istituto di Ricerche Chimiche e Biochimiche “G. Ronzoni”, Via Giuseppe Colombo 81, 20133 Milano, Italy; parafioriti@ronzoni.it (M.P.); elli@ronzoni.it (S.E.); 2 Instituto de Investigationes Químicas (IIQ)-Consejo Superior de Investigaciones Científicas (CSIC), Avenida Americo Vespucio 49, 41092 Sevilla, Spain; juan.munioz@iiq.csic.es (J.C.M.-G.); jonathan.ramirez@iiq.csic.es (J.R.-C.); 3 Department of Biochemistry and Systems Biology, Institute of Systems, Molecular and Integrative Biology, University of Liverpool, Liverpool L69 7ZB, UK; e.a.yates@liverpool.ac.uk; 4 Centre for Glycoscience, Keele University, Newcastle-Under-Lyme ST5 5BG, UK

**Keywords:** ligand-based NMR, STD NMR, DEEP-STD NMR, MD simulations, radial distribution function, molecular recognition, glycosaminoglycan–protein interactions

## Abstract

The interaction of heparin with antithrombin (AT) involves a specific sequence corresponding to the pentasaccharide GlcNAc/NS6S-GlcA-GlcNS3S6S-IdoA2S-GlcNS6S (AGA*IA). Recent studies have revealed that two AGA*IA-containing hexasaccharides, which differ in the sulfation degree of the iduronic acid unit, exhibit similar binding to AT, albeit with different affinities. However, the lack of experimental data concerning the molecular contacts between these ligands and the amino acids within the protein-binding site prevents a detailed description of the complexes. Differential epitope mapping (DEEP)-STD NMR, in combination with MD simulations, enables the experimental observation and comparison of two heparin pentasaccharides interacting with AT, revealing slightly different bound orientations and distinct affinities of both glycans for AT. We demonstrate the effectiveness of the differential solvent DEEP-STD NMR approach in determining the presence of polar residues in the recognition sites of glycosaminoglycan-binding proteins.

## 1. Introduction

Heparin is a long linear negatively charged polysaccharide belonging to the glycosaminoglycan (GAG) family [1]. It is composed of repeating disaccharide units comprising uronic acid (β-D-glucuronic acid or α-L-iduronic acid) (1-4)-linked to α-D-glucosamine. The uronic acids can be 2-O-sulfated, while the glucosamines are N-acetylated or N-sulfated, 6-O-sulfated and, more rarely, 3-O-sulfated [2]. In solution, the glucosamine (GlcN) and glucuronic acid (GlcA) residues adopt a ^4^C_1_ chair conformation, while the iduronic acid (IdoA) residues exist in equilibrium between the ^4^C_1_ chair, ^1^C_4_ chair and ^2^S_0_ skew-boat forms [3,4,5]. The population of each IdoA conformer depends on the degree of sulfation of the iduronic acid and the adjacent glucosamines [6]. Endogenous heparin is located within mast cell granules, where it interacts with histamine, proteases and inflammatory mediators, controlling their storage, retention and activation. Exogenous heparin, derived from porcine and bovine tissues, is used as an anticoagulant drug, due to its ability to bind and regulate the activity of several factors involved in the blood clotting cascade [7]. Specifically, heparin binds and activates antithrombin (AT), accelerating its inhibitory effect on the enzymes of the coagulation system, such as factor IIa (thrombin) and factor Xa. This interaction is crucial in the prevention and treatment of thromboembolic disorders, avoiding the excessive formation of blood clots [8,9,10].

In this context, the study of GAG–protein complexes is fundamental for understanding the molecular mechanisms underlying GAG–protein interactions and, therefore, designing innovative glycomimetic drugs [11,12].

Ligand-based nuclear magnetic resonance (NMR) experiments, including transferred nuclear Overhauser effect spectroscopy (tr-NOESY) and saturation transfer difference (STD) NMR, allow definition of the bioactive conformation and the epitope mapping of the ligand; however, they provide no information about the protein-binding site [13,14,15,16]. A novel multifrequency and multisolvent STD NMR approach, known as differential epitope mapping (DEEP)-STD NMR, was developed by Angulo and co-workers to gain details on the nature of the protein residues (aliphatic, aromatic or polar) involved in the molecular recognition process [17]. This new approach exploits the differences in the ligand epitope mapping that arise from acquiring a pair of STD NMR experiments under two different conditions, such as different frequencies or solvents. The multifrequency STD NMR, conducted by irradiating the aliphatic and aromatic residues of the protein, permits determination of the aliphatic or aromatic nature of the amino acid side chains in contact with the ligand. Ligand protons close to directly irradiated protein residues receive a higher degree of saturation, showing a relative increase in their STD intensities. Multisolvent STD NMR, performed in both D_2_O and H_2_O, allows identification of polar residues in the protein-binding site. In D_2_O, the exchangeable protons of polar residues are replaced by deuterium, which is unable to transfer proton saturation; in H_2_O, these protons may allow an additional transfer of saturation, depending on their exchange rate with the bulk water [18]. If ligand protons are in proximity to slow exchanging polar residues, they receive an extra saturation in the STD NMR experiments conducted in H_2_O, showing a relative increase in their STD intensities.

Theoretical methods, such as atomistic molecular dynamics (MDs) simulations using state-of-the-art force-fields and the 3D structure of GAG–protein complexes obtained by X-ray crystallography or cryogenic electron microscopy (cryo-EM), are crucial to reconstruct the details of the protein residues and intermolecular forces that drive GAG–protein interactions [19,20]. Early MD simulations of a complex between a specific heparin pentasaccharide sequence and AT were run by Grootenhuis et al. (1991) and by Verli et al. (2005), spanning simulation times of less than 1 ns [21,22]. For the first time, they described the contribution of the amino acids of the protein recognition site to ligand binding, identifying that electrostatic forces drive the interaction and sketching the mechanism of allosteric activation. Later, Balogh et al. (2020) applied Gaussian accelerated MD simulations for a deeper analysis of the mechanism of allosteric activation [23]. Sarkar et al. (2016) underlined the solvation energy as one of the key aspects of GAG–protein affinity [24]. Our group applied sub-microsecond MD simulations to unravel the specificity of the interaction between heparin oligosaccharides and AT, successfully complementing the NMR interaction evidence [25,26]. Comprehensive reviews introduce and guide readers on how MD simulations are used to determine local and global structural and conformational properties of GAGs in their unbound and protein-bound states. Almond et al. (2018) gives a wide perspective of theoretical methods for the structural and dynamic aspects of GAGs, underlining the multiscale modelling from mono- to polysaccharides and the timescale from nano- to microseconds [27]. While microsecond MD simulation is a generally desirable target in GAG–protein complex descriptions, some molecular aspects, characterized by short relaxation periods, can also be estimated using sub-microsecond MD simulations. This was verified by the interglycosidic conformations of heparin oligosaccharides in the bound state with FGF-1 by Bojarski et al. (2019) [28].

Several studies have been performed by our group to elucidate the intricate molecular recognition processes underlying the interactions between GAGs and their respective receptors, with a specific focus on the interaction between heparin and AT. Within this context, our investigations revealed a noteworthy phenomenon wherein the IdoA2S unit of the heparin pentasaccharide, a region specifically recognized by AT, undergoes a significant conformational shift toward the ^2^S_0_ skew-boat conformation upon binding [29]. This conformational change was found to be independent of the sulfation state of the iduronate unit and the specific position of the pentasaccharide along the heparin chain [25,30]. Despite the valuable insights gained from these studies, the absence of experimental data concerning the molecular contacts between the ligand and the amino acids within the protein-binding site has prompted the exploration of advanced techniques. In this regard, the application of DEEP-STD NMR emerges as a promising avenue.

**Figure 1:**
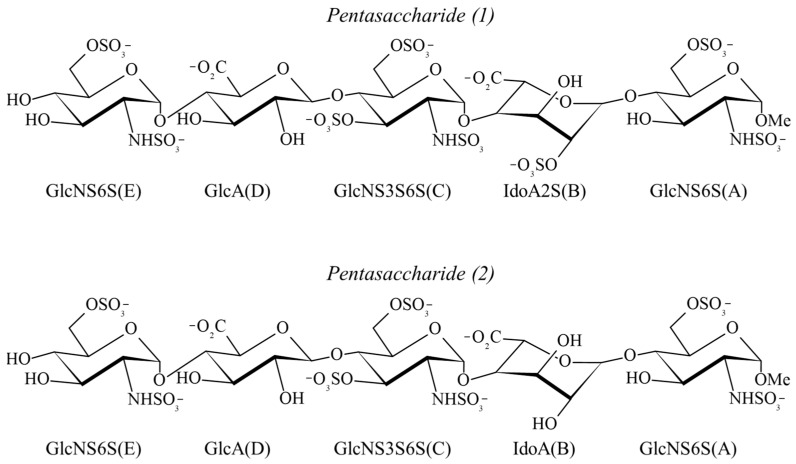
Chemical structure of the heparin pentasaccharides selected as AT binders. The ligand units are depicted in their prevalent conformation in the unbound state: the GlcN and GlcA units adopt the ^4^C_1_ form, while the IdoA2S and IdoA units are in equilibrium between the ^1^C_4_ and ^2^S_0_ forms.

In this work, we explored the ability of DEEP-STD NMR, carried out in different solvents (D_2_O and H_2_O), to identify the presence of polar residues in the heparin-binding site of AT, which is mainly constituted by arginine (R) and lysine (K) residues (K11, R13, R46, R47, K114, K125 and R129) [31,32,33]. It is known that AT binds to heparin recognizing a specific pentasaccharide sequence [D-GlcNS6S α (1-4) D-GlcA β (1-4) D-GlcNS3S6S α (1-4) L-IdoA2S α (1-4) D-GlcNS6S α (1-4)] present in about 30% of its chains [34,35], even if some degeneracy of this sequence has been observed in limited cases [25,26]. Therefore, two structurally related heparin pentasaccharides (1) and (2) were selected for comparison as ligand probes (Figure 1).

The structure of pentasaccharide (2) differs from that of pentasaccharide (1), which corresponds to the heparin sequence that is specifically recognized by AT, only through the absence of the 2-O-sulfate group in the iduronate unit (Figure 1). Owing to the absence of the 2-O-sulfate group in the iduronic acid unit, pentasaccharide (2) possesses a lower affinity than pentasaccharide (1) [36,37,38]. The binding of pentasaccharide (1) to AT is well-described: in the first stage, the stiff non-reducing trisaccharide of pentasaccharide (1), attracted by the positively charged surface of the AT-binding site, induces a protein conformational change (AT activation) that allows, in the second stage, interaction with the flexible reducing disaccharide [39,40,41]. This phase is also assisted by the conformational flexibility of the iduronic acid unit, which adopts a pure ^2^S_0_ skew-boat conformation upon binding, enhancing the contacts. Since the reducing disaccharide of pentasaccharide (1) interacts with AT in an area of the binding pocket with the highest density of arginine residues, we expected to observe a relative increase in the STD intensities of the ligand protons belonging to this moiety in the STD NMR experiment conducted in H_2_O. The same behavior was also hypothesized for pentasaccharide (2), whose binding mode should be similar, albeit not exactly analogous, to that of pentasaccharide (1). Additionally, differences in the conformations of pentasaccharides (1) and (2) in the AT-bound state were investigated by tr-NOESY experiments combined with docking and explicit solvent MD simulations covering 1 µs.

Finally, the methodology based on the reduced relaxation matrix (RedMat) was carried out to simulate the theoretical binding epitopes and to compare them with the corresponding experimental ones in order to validate the MD simulation trajectories of the two complexes [42].

## 2. Results

### 2.1. Comparison of the Interactions of Pentasaccharides (1) and (2) with AT Using STD and DEEP-STD NMR

**Figure 2:**
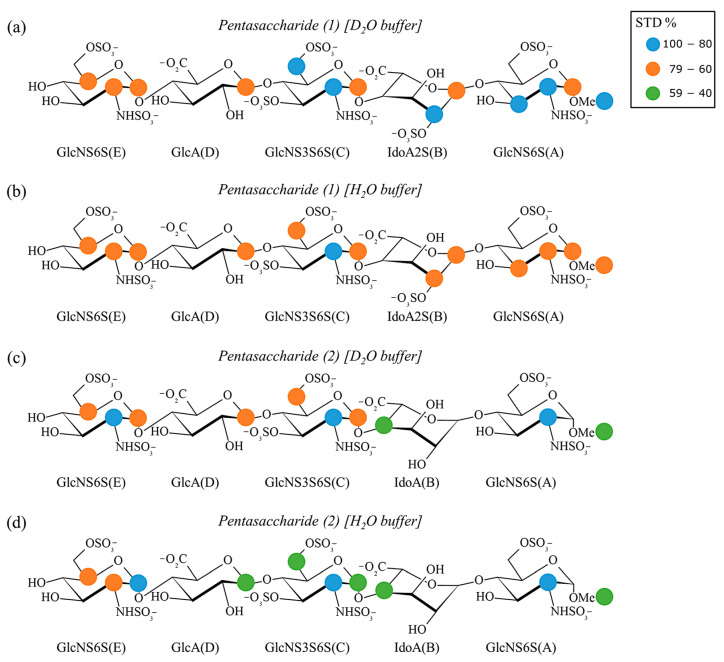
Ligand epitope mapping of pentasaccharides (1) and (2) for their interaction with AT. Panels (**a**,**b**): Binding epitope maps of pentasaccharide (1) in D_2_O and H_2_O. Panels (**c**,**d**): Binding epitope maps of pentasaccharide (2) in D_2_O and H_2_O.

For each complex, two samples, one in heavy water buffer (D_2_O) and one in standard aqueous buffer (H_2_O), were prepared to perform differential solvent DEEP-STD NMR analysis. The STD NMR spectra were recorded at 0.5 s saturation time (Figures S1–S4). The absolute and relative STD percentages were calculated as described in the experimental section (Tables S1 and S2), and the binding epitope maps were obtained in both D_2_O and H_2_O conditions (Figure 2).

For pentasaccharide (1), the epitope mapping in D_2_O showed that H2C, H6″C, H2B, H2A, H3A and OCH_3_(A) receive the strongest saturation (80–100%), while H1E, H2E, H5E, H1D, H1C, H1B and H1A are slightly less affected by the saturation transfer (60–80%) (Table S1 and Figure 2a). The same analysis in H_2_O found that all the mentioned protons present an STD intensity in the range of 60–80%, except for H2C, whose STD value is 100% (Table S1 and Figure 2b). Overall, considering the experimental error, the STD NMR results suggested a consistent binding mode of pentasaccharide (1) in both solvents. However, two significant observations emerged from the binding epitope maps (Figure 2a,b). Firstly, the STD NMR analysis in D_2_O revealed remarkable interactions between the reducing disaccharide and the protein. This indicates that STD NMR provides valuable insights into the final conformation of the bound ligand after the two steps of the recognition process described in the literature, leading to close contacts with the reducing disaccharide [39,40]. Secondly, the relative STDs were lower in H_2_O than in D_2_O, suggesting that the presence of exchangeable polar protons amplifies the relaxation-based decrease in the saturation transfer to the ligand protons (saturation leakage).

The epitope map of pentasaccharide (2) in D_2_O showed that the STD intensity is strong (80–100%) for H2E, H2C and H2A, medium (60–80%) for H1E, H5E, H1D, H1C and H6″C and low (40–60%) for H4B and OCH_3_(A) (Table S2 and Figure 2c). The same evaluation in H_2_O displayed strong STD enhancements for H1E, H2C and H2A, medium STD effects for H2E and H5E and low STD percentages for H1D, H1C, H6″C, H4B and OCH_3_(A) (Table S2 and Figure 2d). Because of the superimposition between the signals of the anomeric protons of IdoA(B) and GlcNS6S(A), these could not be integrated separately; nevertheless, the STD NMR spectra indicate that they are involved in the interaction (Figures S3 and S4). In the case of pentasaccharide (2), the binding epitope map in D_2_O showed higher relative STDs for the non-reducing trisaccharide, which might indicate that the second step of binding is not as efficient and, therefore, AT does not hold the reducing disaccharide toward its surface as tightly as pentasaccharide (1). This is in agreement with the lower affinity of pentasaccharide (2) and supports the contention that the absence of the 2-O-sulfate group in the iduronic acid unit of pentasaccharide (2) confers the higher mobility of the IdoA(B)-GlcNS6S(A) disaccharide at the reducing end [36]. In fact, the comparison of the binding epitopes between the pentasaccharide (1)-AT and pentasaccharide (2)-AT complexes in D_2_O revealed that OCH_3_(A) receives significantly lower saturation in the pentasaccharide (2)-AT complex. Again, the impact of exchangeable polar protons in the H_2_O sample globally reduces the relative STDs of the ligand, as in the case of pentasaccharide (1). An additional difference between the binding epitope maps of both pentasaccharides was observed for H6″C: the lower STD enhancement characterizing this group in the pentasaccharide (2)-AT system suggests a weaker contact between the 6-O-sulfate group of GlcNS3S6S(C) and R46 (as it will be shown later in the text).

Next, the application of the differential solvent DEEP-STD NMR led to an interesting observation from the comparison of the total levels of saturation (i.e., the sum of the absolute STD intensities) achieved in D_2_O with that recorded in H_2_O. Notably, this analysis revealed that the saturation transferred to pentasaccharide (1) was lower in D_2_O than in H_2_O (15.16% < 19.73%), whereas, on the contrary, the saturation received by pentasaccharide (2) was higher in D_2_O than in H_2_O (19.50% > 15.94%) (Tables S1 and S2). This difference correlates with the distinct affinity of these pentasaccharides for AT. Indeed, high affinity determines a long residence time of the ligand within the protein pocket, which leads to greater protection of the protein polar protons interacting with the ligand from their exchange with the bulk water. This enhances their contribution to the saturation transfer to the ligand, increasing the total saturation available for the transfer to the ligand protons. In this case, the protection is higher when pentasaccharide (1) is bound to AT, due to its higher affinity for the protein. In this way, the results establish that the differential solvent DEEP-STD NMR data comparison of similar ligands to a given protein receptor is useful to explain differences in affinity. While it is challenging to directly correlate changes in absolute STD intensities with differences in affinity, as the observation of STD signals can be influenced by varying kinetics, detecting an increase in the total saturation acquired by a ligand in H_2_O in comparison to D_2_O would suggest a high affinity interaction (within the range of the STD NMR observation). In contrast, ligands with medium or low affinity (within the range of the STD NMR observation) are expected to show a decrease in the total saturation when changing from D_2_O to H_2_O.

**Figure 3:**
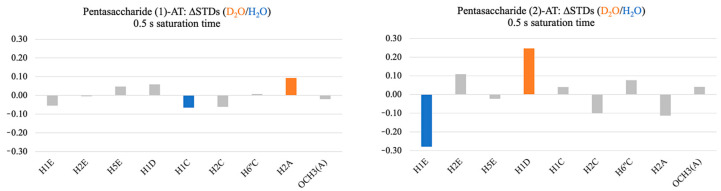
Differential epitope mapping (D_2_O/H_2_O) of both pentasaccharides (1) and (2) in complex with AT. ΔSTD histograms: protons with the strongest positive ΔSTDs are in orange; protons with the highest negative ΔSTDs are in blue.

From the differential solvent DEEP-STD NMR experiments, we carried out an analysis of the DEEP-STD factors (∆STDs). To compare equally the DEEP-STD NMR data collected from the analyzed systems, only the STD NMR signals in common between the two complexes were selected. Interestingly, the two complexes showed significantly different patterns in the DEEP-STD NMR data (Table S3 and Figure 3).

The pentasaccharide (1)-AT complex displayed very small and probably not significant DEEP-STD factors (Table S3 and Figure 3). In contrast, the differential epitope map of the pentasaccharide (2)-AT complex exhibited stronger negative and positive ∆STDs for H1E and H1D, respectively (Table S3 and Figure 3). The relative decrease in the ∆STD characterizing H1E in D_2_O suggests proximity to slow exchanging polar residues (i.e., arginine), while the relative increase in the ∆STD belonging to H1D in D_2_O indicates that this ligand proton is close to fast exchanging polar residues (i.e., lysine) and/or non-exchangeable residues. The similarity of the binding epitopes, despite displaying significantly different DEEP-STD NMR patterns, was intriguing, prompting the use of MD simulations to deepen our understanding of the observed differences.

### 2.2. Comparative Analysis of the Conformations of Pentasaccharides (1) and (2) in the Unbound and AT-Bound States by ^3^J_H-H_ Coupling Constants and NOESY/tr-NOESY Experiments

Three-bond proton–proton coupling constants (^3^J_H-H_) of pentasaccharides (1) and (2) were measured to define the conformation of the sugar rings in the free state (Tables S4 and S5). The analysis of the ^3^J_H-H_ values showed that the glucosamines [GlcNS6S(E, C, A)] and the glucuronic acid [GlcA(D)] in both pentasaccharides adopt a ^4^C_1_ chair conformation, while the iduronic acid in pentasaccharide (1) [IdoA2S(B)] exists in equilibrium between the ^1^C_4_ chair (36%) and ^2^S_0_ skew-boat (64%) conformations, according to Ferro et al. (1990) [6,43]. Unfortunately, the overlapping of the IdoA(B) signals did not permit the same analysis in pentasaccharide (2) to be performed. The NOE results confirmed the conformational data obtained from the ^3^J_H-H_ analysis for both pentasaccharides in the unbound state. NOESY and tr-NOESY experiments were carried out to describe the intra-residue and interglycosidic conformational changes in the two glycans after binding (Figures S5 and S6). A significant increase in the intensity of the cross-peaks of the tr-NOESY spectra (in the presence of the protein) compared to the NOESY spectra (free ligands) was observed, providing proof of interaction (Tables S6–S10). The conformation of the iduronic acid in pentasaccharides (1) and (2) in the free state and upon AT-binding was investigated by comparing the NOE and tr-NOE data. Since the iduronic acid exhibits distinct H5-H2 distances in the ^1^C_4_ chair and ^2^S_0_ skew-boat conformations (4.0 Å and 2.4 Å, respectively), the ratio between the H5-H2 and H5-H4 NOEs permits estimation of its prevalent form. Unfortunately, the partial overlapping of the H5B-H4B cross-peak in the NOESY and tr-NOESY spectra of pentasaccharide (1) and pentasaccharide (1)-AT complex, respectively, with the H5B-H3B signal (generated by spin diffusion) did not allow the conformer populations of IdoA2S(B) in the unbound and bound states to be confirmed. Nevertheless, it is well described that the iduronic acid unit belonging to the pentasaccharide (1) structure approaches a pure ^2^S_0_ form upon AT-binding [6,29,44]. The interaction between pentasaccharide (2) and AT changes the H5B-H2B/H5B-H4B NOE ratios of IdoA(B) from 0.3 to 0.7, revealing that the conformation of this moiety, which prevalently adopts the ^1^C_4_ chair conformation in the free state, is only partially shifted toward the AT-binding ^2^S_0_ skew-boat form in the bound state (Table S8). This result supports the reduced involvement of the reducing end moiety [IdoA(B)-GlcNS6S(A)] of pentasaccharide (2) in the binding to AT observed by STD NMR. The comparison of the interglycosidic NOEs and tr-NOEs, defined for both glycans in the unbound and bound states, underlined that the GlcA(D)-GlcNS3S6S(C) linkage is the only part of the glycosidic backbone that undergoes a significant conformational change upon AT-binding (the H1D-H6″C/H1D-H4C NOE ratios change from 1.6–1.1 to 1.1–0.5) (Tables S9 and S10).

### 2.3. Comparison of the Interactions of Pentasaccharides (1) and (2) with AT by Molecular Docking

The orientation that the two ligands assume in the protein-binding site was confirmed by molecular docking. The evaluation of the obtained poses was carried out considering the binding energy (GlideScore) and the root-mean square distance (RMSD) from the position of the reference pentasaccharide co-crystallized with AT (PDB ID: 1AZX) [39]. For the pentasaccharide (1)-AT complex, few poses were identified. In particular, the best pose, characterized by a GlideScore of −9.0 Kcal/mol and RMSD of 2.0 Å, is very similar to the X-ray orientation (PDB ID: 1AZX). For the pentasaccharide (2)-AT complex, the best pose is defined by a GlideScore of −9.5 Kcal/mol and RMSD of 3.0 Å. In contrast to the pentasaccharide (1)-AT system, several poses were collected when pentasaccharide (2) was docked on AT, some of them characterized by shifts in the glucosamine at the reducing end [GlcNS6S(A)]. In general, the docking results confirmed what was already known about the binding mode of heparin oligosaccharides to AT: the pentasaccharide sequence, here represented by the structures (1) and (2), specifically binds AT in the active site formed by helix-D, assuming a single orientation where its reducing end points toward the bent of the helix. Interestingly, the difference in the binding affinity between the two pentasaccharides can be correlated to the few poses obtained for pentasaccharide (1), implying the tighter binding of this ligand to the protein, against the higher number of structures collected for pentasaccharide (2), indicating greater mobility of this compound in the binding pocket. As docking calculations deal with rigid structures of the protein receptor, we next decided to carry out MD simulations of the complexes.

### 2.4. Investigation of the Structural Features of the Pentasaccharide (1)-AT and Pentasaccharide (2)-AT Complexes by MD Simulations

**Figure 4:**
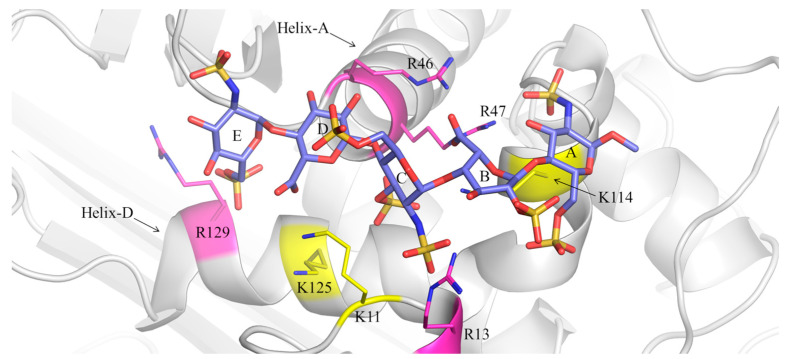
Selected geometry of the pentasaccharide (1)-AT complex (600 ns simulation time). Pentasaccharide (1) is represented by purple, red, blue and yellow tubes corresponding to carbon, oxygen, nitrogen and sulfur atoms; AT is reported as grey ribbons; arginine (R) residues are shown as fuchsia ribbons and thin fuchsia and blue tubes representing carbon and nitrogen atoms; lysine (K) residues are displayed as yellow ribbons and thin yellow and blue tubes indicating carbon and nitrogen atoms. Letters from E to A indicate the residues of pentasaccharide (1) as in Figure 1. Helix-A and Helix-D in AT are labelled.

**Figure 5:**
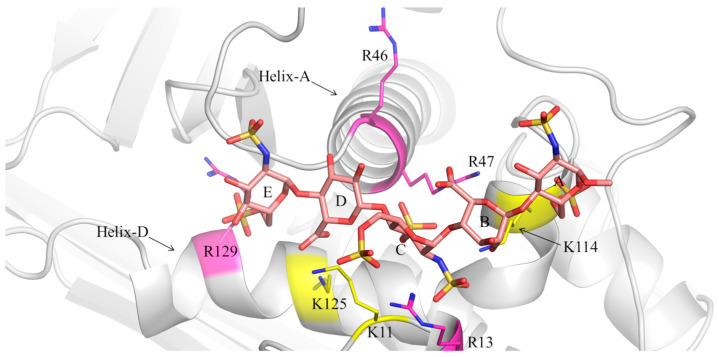
Selected geometry of the pentasaccharide (2)-AT complex (600 ns simulation time). Pentasaccharide (2) is represented by pink, red, blue and yellow tubes corresponding to carbon, oxygen, nitrogen and sulfur atoms; AT is reported as grey ribbons; arginine (R) residues are shown as fuchsia ribbons and thin fuchsia and blue tubes representing carbon and nitrogen atoms; lysine (K) residues are displayed as yellow ribbons and thin yellow and blue tubes indicating carbon and nitrogen atoms. Letters from E to A indicate the residues of pentasaccharide (2) as in Figure 1. Helix-A and Helix-D in AT are labelled.

The initial 3D structures of the pentasaccharide (1)-AT and pentasaccharide (2)-AT complexes were built based on the pentasaccharide-AT complex obtained by X-ray diffraction (PDB ID: 1AZX). The latter was found to be comparable to the docking poses characterized by the best GlideScore values. Both complexes were simulated for 1 μs in explicit solvent condition, sampling the isothermal-isobaric (NPT) ensemble. Figure 4 and Figure 5 depict two snapshots extracted from the MD simulation trajectories at 600 ns.

The analysis of the RMSD showed that pentasaccharide (1) remains stable in the AT-binding pocket for the entire simulation time, while pentasaccharide (2) in the AT-bound state presents higher mobility that correlates to its lower affinity for the protein, even if no drift indicating early unbinding of this glycan was observed over the timescale (Figure S7). The root-mean square fluctuation (RMSF) analysis allowed the flexibility observed for pentasaccharide (2) within the AT-binding site to be correlated with the fluctuations of GlcNS6S(A) at its reducing end. Specifically, the comparison of the RMSF values calculated for the ligand units revealed that GlcNS6S(A) exhibits higher fluctuations in pentasaccharide (2) compared to its respective unit in pentasaccharide (1) and all other units (Figure S8a). Furthermore, a similar evaluation conducted on key amino acids of the AT active site displayed higher mobility of the R13 and R129 side chains when pentasaccharide (1) binds to AT (Figure S8b).

The known flexibility of the iduronate moiety in the AT-bound state was investigated plotting the distance between the H5 and H2 protons of IdoA2S(B) and IdoA(B) (Figure S9) [6,45,46,47,48]. This analysis revealed that IdoA2S(B) in the pentasaccharide (1)-AT complex presents a higher percentage of the ^2^S_0_ form than IdoA(B) in the pentasaccharide (2)-AT complex, in agreement with what was observed by NMR.

**Figure 6:**
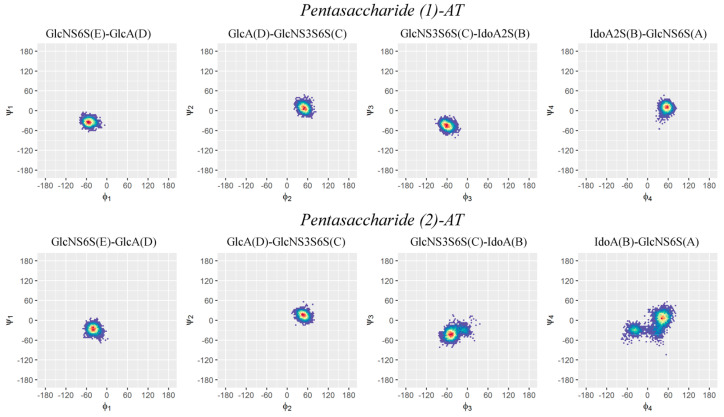
Ramachandran plots of the glycosidic dihedral angles φ_i_/ψ_i_ of pentasaccharides (1) and (2) in the AT-bound state. A density color map is superposed on each Ramachandran plot; the color gradient (blue to red) is proportional to the density of the sampled states φ_i_/ψ_i_ and qualitatively predicts the preferred conformation of each glycosidic linkage.

The conformations of the glycosidic linkages of pentasaccharides (1) and (2) in the AT-bound state were described by Ramachandran plots, in which a color gradient density map allowed the most populated states to be localized (Figure 6).

**Figure 7:**
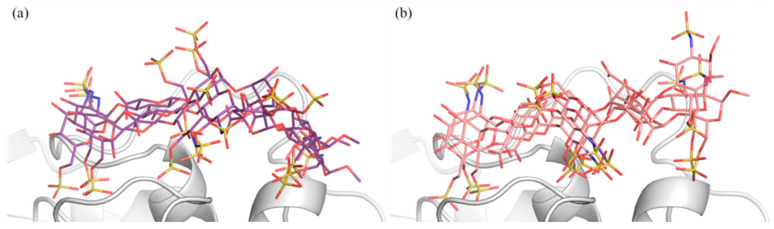
Pentasaccharide (1) [panel (**a**)] and pentasaccharide (2) [panel (**b**)] interacting with AT. For each pentasaccharide, three structures were selected from the corresponding MD simulation. These snapshots were sampled considering the most populated states of each glycosidic dihedral angle φ_i_/ψ_i_. Pentasaccharide (1) is represented by purple, red, blue and yellow tubes corresponding to carbon, oxygen, nitrogen and sulfur atoms; pentasaccharide (2) is depicted as pink, red, blue and yellow tubes indicating carbon, oxygen, nitrogen and sulfur atoms; AT is reported as gray ribbon.

The conformational analysis revealed that pentasaccharide (1) is characterized by a single populated state for each dihedral angle, while pentasaccharide (2) exhibits greater conformational mobility at the reducing end (Figure 6, Figure 7 and Figure S10).

Specifically, it unveiled that the glycosidic bond between IdoA(B) and GlcNS6S(A) in pentasaccharide (2) presents two populated φ_i_/ψ_i_ states. The most populated states for each dihedral angle of the two systems and the relative population percentages of the dihedral angle between IdoA(B) and GlcNS6S(A) are reported in Table 1.

MD poses based on these interglycosidic dihedral angles illustrate different orientations of the reducing end, consistent with the observed increased mobility of this substructure within the pentasaccharide (2)-AT complex, as confirmed by the RMSD and RMSF spectra (Figure S10).

The dynamic ensembles derived from MD simulations were also subjected to comprehensive clustering analysis to discern groups of structures sharing similar conformational features. In the case of pentasaccharide (1), the clustering analysis unveiled a single dominant cluster, indicating a homogeneous conformational ensemble (Figure S11a,b). Conversely, pentasaccharide (2) exhibited greater structural diversity, resulting in the identification of two principal clusters corresponding to two distinct conformers (Figure S11c,d). The dihedral angles of the structures obtained by cluster analysis (Table 1) were compared against those defined by Ramachandran plots (Table S11), demonstrating remarkable agreement. Interestingly, a comparison of the dominant conformers of pentasaccharides (1) and (2) highlighted variations in their relative positions within the AT-binding site. More precisely, the pentasaccharide (1) structure is slightly translated toward helix-A, and its IdoA2S(B)-GlcNS6S(A) moiety bends over the N-terminal end of helix-D, allowing tighter contacts (Figures S10 and S11).

The interaction between pentasaccharide (1) and AT is characterized by manifold contacts between the negatively charged groups of the oligosaccharide, such as sulfate and carboxylic groups, and the positively charged side chains of the arginine (R) and lysine (K) residues that characterize the AT-binding site [49]. According to previous studies, the main electrostatic interactions are the following: [GlcNS6S(E)(6S)]-K125, [GlcNS6S(E)(6S)]-R129, [GlcA(D)(COO^−^)]-K125, [GlcNS3S6S(C)(NS)]-R13, [GlcNS3S6S(C)(NS,3S)]-K114, [GlcNS3S6S(C)(6S)]-R46, [IdoA2S(B)(2S)]-R13, [IdoA2S(B)(COO^−^)]-R46, R47 and K114, [GlcNS6S(A)(NS)]-R46 and R47, [GlcNS6S(A)(6S)]-R13 and K114 (Figure 4). These contacts, averaged over the production stage of MD simulations, are shown for both complexes in Table 2; analogous distances in 1AZX are also reported for comparison purposes.

The contacts between the pentasaccharide and AT in 1AZX are preserved in the simulated pentasaccharide (1)-AT complex, with only minor differences at the level of GlcA(D)(COO^−^)-K125, GlcNS3S6S(C)(NS)-K114 and GlcNS6S(A)(6S)-R13.

Monitoring these interactions in the pentasaccharide (2)-AT complex showed that pentasaccharide (2) loses some of these contacts with key amino acids of the AT-binding site (Figure 5 and Figures S12–S16). In particular, the interactions [GlcNS6S(E)(6S)]-K125, [GlcNS3S6S(C)(6S)]-R46, [IdoA(B)(COO^−^)]-R46, R47 and K114, [GlcNS6S(A)(NS)]-R46 and R47 and [GlcNS6S(A)(6S)]-R13 are weaker in the pentasaccharide (2)-AT complex, according to the longer distances between the aforenamed contacts (Table 2 and Figures S12a, S14d, S15b–d and S16a–c). Interestingly, the differences in the contacts of pentasaccharides (1) and (2) and AT are less evident at the level of the GlcNS6S(E)-GlcA(D)-GlcNS3S6S(C) trisaccharide and significant at the reducing-end IdoA(B)-GlcNS6S(A), supporting the distinct positions of these glycans in the active site of AT (Table 2, Figures S10 and S11). These findings highlight a correlation between the greater mobility observed at the reducing terminal of pentasaccharide (2), as revealed by the RMSF of GlcNS6S(A), and the weaker contacts between its IdoA(B)-GlcNS6S(A) disaccharide and key amino acids within the AT-binding site (Table 2, Figures S8a, S15b–d and S16a–c). In contrast, the electrostatic interaction between the carboxylic group of GlcA(D) and K125 becomes particularly relevant in the binding of pentasaccharide (2) to AT (Table 2 and Figure S13). Moreover, the pentasaccharide (1)-AT and pentasaccharide (2)-AT complexes also differ in the position of R13, which is in the former complex between the 2-O-sulfate group of IdoA2S(B) and the 2-N-sulfate group of GlcNS3S6S(C), while it moves in the second complex between 2-N-sulfate and 6-O-sulfate groups of GlcNS3S6S(C) (Table 2, Figure 4 and Figure 5). This different position of R13 also correlates with the higher stability of its contacts with GlcNS3S6S(C) in the pentasaccharide (2)-AT system (Figure S14a), as also evinced by the lower RMSF values for these moieties (Figure S8a,b).

These distinct binding modes predicted for pentasaccharides (1) and (2) collectively correlate with the higher affinity of pentasaccharide (1) to AT, as determined by the Poisson Boltzmann free energy of binding (${∆G}_{PB}^{bind}$) of −98.9(7) Kcal/mol compared to −71.8(7) Kcal/mol found for pentasaccharide (2).

### 2.5. Comparison of the Solvation Properties at the Binding Interfaces of the Pentasaccharide (1)-AT and Pentasaccharide (2)-AT Complexes by MD Simulations

The influence of water on the binding of pentasaccharides (1) and (2) to AT was evaluated using the radial distribution function [g(r)]. This analysis allows characterization of the size and distribution of the hydration shells surrounding selected protein residues or ligand units by determining the radial density of water molecules at a distance (r) from a reference atom (or set of atoms) [50]. For such polar ligands, the local concentration and organization of water molecules are assumed to affect the protein to ligand transfer of saturation and/or dispersion of magnetization in the STD NMR experiment.

The solvation analysis of the protein residues belonging to the heparin-binding site of AT showed that the hydration networks around the side chains of R13, K114 and R129 and characterizing the side chains of K11, R47 and K125 determine different shielding effects on the pentasaccharide (1)-AT and pentasaccharide (2)-AT complexes, respectively, due to the distinct relative positions that the two ligands assume in the protein-binding pocket (Figure 4, Figure 5, Figures S10, S11 and S17). In contrast, the distribution of water molecules surrounding R46 is similar in both complexes (Figure S17c). The same evaluation, carried out for the ligand units, revealed that GlcNS6S(E), GlcA(D) and GlcNS6S(A) are characterized by comparable shells of water molecules in both systems, while GlcNS3S6S(C) and IdoA2S(B)/IdoA(B) are more solvated in the pentasaccharide (2)-AT complex, indicating greater distance of these units from the protein surface that permits water molecules to permeate the binding site under the ligand (Figure S18).

To explain the differences in the DEEP-STD NMR data collected for the studied systems, this kind of analysis was also performed for the ligand protons with a significant ∆STD factor (Figure 3). A negative ∆STD was obtained for H1E only in the pentasaccharide (2)-AT complex, indicating the proximity of this proton to polar groups (i.e., arginine) in the binding pocket of AT (Table S3 and Figure 3). Although with small differences, the binding epitope maps of both ligands do not support large binding mode differences that could explain such observation (Figure 2). Differences in the solvation of this proton were clearly predicted by the MD simulations, which explain the NMR experimental result. Only in the pentasaccharide (1)-AT complex, H1E showed a well-defined solvation shell (Figure S19a). The presence of structured water around the ligand protons can induce very efficient magnetization leakage by chemical exchange of the solvating molecules with the bulk water in solution. Under these circumstances, in the pentasaccharide (1)-AT complex, the enhanced saturation in H_2_O, due to the presence of the side chain of R129 proximal to GlcNS6S(E), is compensated by such an efficient saturation sink (Table S1 and Figure S19a). In contrast, the solvation shell of H1E is missing in the pentasaccharide (2)-AT complex, where this proton is located proximal to the backbone of the threonine residue T44 (Figure 5 and Figure S19a). Consequently, H1E in the pentasaccharide (2)-AT complex efficiently receives an extra saturation transfer from the nearby arginine residue R129 when the STD NMR experiment is conducted in H_2_O (Figures S12a and S19a). Additionally, a significant positive ∆STD was obtained for H1D only in the pentasaccharide (2)-AT complex (Table S3 and Figure 3). Again, this suggests efficient saturation leakage that correlates very well with the solvation shells of the nearby lysine residues K11 and K125 that show higher local concentrations of water molecules and closer contacts to GlcA(D) in the pentasaccharide (2)-AT complex (Figure S17a,f). More precisely, the average distances between H1D and the nitrogen (+)NH_3_ of the side chains of K11 and K125 are 4.8 Å and 8.1 Å in the pentasaccharide (2)-AT complex, respectively, and 5.8 Å and 10.4 Å in the pentasaccharide (1)-AT complex (see also Figure 4 and Figure 5).

In summary, the absence of the 2-O-sulfate group of IdoA(B) induces changes in the solvation properties around the protein–ligand interfaces, as predicted by MD simulations and experimentally supported by the DEEP-STD NMR experiments. Thus, although the binding epitope maps indicate that the binding modes of both ligands to AT are rather similar, these changes in water distribution around the binding pocket correlate well with their different affinities (Figure 2).

### 2.6. Model Validation by RedMat

The RedMat analysis was carried out for validating the 3D models built by MD simulations [42]. The theoretical binding epitope was estimated using the MD trajectories of the pentasaccharide (1)-AT and pentasaccharide (2)-AT complexes (Tables S12 and S13). Figures S20 and S21 show the evolution of the R-factor over the production stage of MD simulations. An average R-factor of 0.26 (with a standard deviation of 0.03) for the pentasaccharide (1)-AT system and 0.29 (with a standard deviation of 0.03) for the pentasaccharide (2)-AT system revealed good agreement between the theoretical and experimental binding epitopes.

## 3. Discussion

In this study, recent advanced ligand-based NMR techniques and computational methods were applied to compare the interaction between two structurally related heparin pentasaccharides and AT. The pentasaccharides (1) and (2) differ only by one sulfate group in their iduronate unit and are characterized by distinct affinities for AT and biological activities [36,37,38]. Their molecular recognition process with AT was dissected by STD NMR experiments in different solvents (differential solvent DEEP-STD NMR) complemented by microsecond-long explicit solvent MD simulations.

The STD binding epitopes of the two pentasaccharides were compared, revealing a similar binding mode for the non-reducing-end trisaccharide, but reduced involvement of the reducing-end disaccharide in the binding of pentasaccharide (2) with AT. These findings are consistent with observations from previous studies involving heparin oligosaccharides [25]. NMR and MD simulations showed that IdoA2S(B) in the pentasaccharide (1)-AT complex presents a higher percentage of the AT-binding ^2^S_0_ skew-boat form than IdoA(B) in the pentasaccharide (2)-AT complex. This observation supports the synergistic contribution to the affinity between heparin oligosaccharides and AT of the ^2^S_0_ skew boat conformation of the iduronic acid and helix-D that accommodates this moiety [29,48,51]. Moreover, the MD simulations, the analysis of the interglycosidic dihedral angles and the cluster analysis predicted that pentasaccharide (1) bound to AT is characterized by a single φ_i_/ψ_i_ geometry, while pentasaccharide (2) presents two dominant clusters with different interglycosidic conformations at the reducing-end moiety and different relative positions compared to that of pentasaccharide (1) bound to AT. This description correlates with the higher conformational mobility of the reducing-end moiety of pentasaccharide (2), as indicated by its RMSF spectrum and its higher contact distances with helix-D, due to the lower affinity of this glycan for the receptor. The evaluation of the electrostatic interactions indicated that the contacts [GlcNS6S(E)(6S)]-K125, [GlcNS3S6S(C)(6S)]-R46, [IdoA(B)(COO^−^)]-R46, R47 and K114, [GlcNS6S(A)(NS)]-R46 and R47 and [GlcNS6S(A)(6S)]-R13 are weaker in the pentasaccharide (2)-AT complex than in the pentasaccharide (1)-AT complex. Moreover, the residue R13, which in the pentasaccharide (1)-AT complex approaches the 2-O-sulfate group of IdoA2S(B) and the 2-N-sulfate group of GlcNS3S6S(C), moves between 2-N-sulfate and 6-O-sulfate groups of GlcNS3S6S(C), also showing reduced mobility, when pentasaccharide (2) binds AT. Consequently, the estimated Poisson Boltzmann free energy of binding is smaller when pentasaccharide (2) binds AT. These results were strengthened by observing that the MD simulations trajectories of the two complexes exhibited the binding epitopes in agreement with the corresponding experimental data, as depicted by RedMat analysis. The differential solvent DEEP-STD NMR study combined with MD simulations showed an intriguing difference in the DEEP-STD NMR responses of the two glycans, strongly suggesting that the bound states of pentasaccharides (1) and (2) within the AT-binding site are characterized by a different local distribution of water molecules. In the pentasaccharide (2)-AT complex, a negative DEEP-STD factor was observed for the anomeric proton of the GlcNS6S(E) unit due to an additional transfer of saturation originating from the nearby arginine residue R129; while a positive DEEP-STD factor was obtained for the anomeric proton of GlcA(D) because of saturation leakage caused by the neighboring lysine residues K11 and K125. The difference in the DEEP-STD response in the pentasaccharide (1)-AT complex arises from distinct solvation patterns of specific ligand units and protein residues.

In this work, two structurally related ligands are shown to have rather similar modes of binding but significantly different DEEP-STD NMR responses, arising from their differential solvation properties in the bound state. We propose that, for extremely polar binding interfaces, such as those involved in GAG–protein interactions, the combination of differential solvent DEEP-STD NMR and MD simulations enables researchers to disentangle their different contributions to the DEEP-STD NMR results, which can provide valuable information concerning solvation to correlate with differences in ligand affinities.

## 4. Materials and Methods

### 4.1. Ligands and Protein

Antithrombin (AT) (Kybernin P) was purchased from CSL Boehringer (Milano, Italy). The product, containing AT and some excipients (amino acetic acid, sodium citrate, sodium chloride and hydrochloric acid/sodium hydroxide), was purified by affinity chromatography using a HiTrap Heparin HP column (Cytiva) Fisher Scientific (Madrid, Spain). After equilibrating the column with 10 column volumes (CVs) of binding buffer (10 mM phosphate buffer pH 7.4 with 150 mM NaCl), the sample was loaded using a syringe fitted to the luer connector. Subsequently to a column washing step with 5 to 10 CV of binding buffer, the protein was eluted with 5 to 10 CV of elution buffer (10 mM phosphate buffer pH 7.4 with 3 M NaCl). The purified product was loaded onto an Amicon^®^ Ultra-4 Centrifugal Filter (10 kDa membrane, 4 mL, Merck Life Science S.L.U. Madrid, Spain) for desalting and buffer exchange. The final concentration was determined by the Thermo Scientific (Madrid, Spain) NanoDrop Microvolume UV–Vis spectrophotometer, using direct absorbance at 280 nm, which is mostly due to the aromatic chains on tryptophan and tyrosine. The purity was assessed by the matrix-assisted laser desorption/ionization-time of flight (MALDI-TOF) mass spectrometer.

### 4.2. NMR Experiments

NMR experiments were performed using a 600 MHz NMR spectrometer featuring a cryoprobe.

For the characterization of pentasaccharides (1) and (2), ^1^H, ^1^H-^1^H TOCSY and ^1^H-^13^C HSQC spectra were acquired. Tables S14 and S15 report the ^1^H and ^13^C chemical shift assignments of the two pentasaccharides, while Figures S22 and S23 show their ^1^H-^13^C HSQC spectra.

For the acquisition of the STD NMR spectra, the samples were prepared dissolving both ligand and protein in 10 mM phosphate buffer pH 7.4 with 150 mM NaCl (D_2_O or H_2_O). Additionally, 0.3 mM deuterated EDTA was added in the H_2_O buffer. The final concentrations were 2 mM for the ligand and 50 µM for the protein (ligand/protein ratio of 50:1). The STD NMR spectra were recorded using the pulse sequences stddiff.3 and stddiffesgp.3 for the sample in D_2_O and H_2_O, respectively. They were acquired at 298 K using 0.5 s as saturation time. A 10 ms spin-lock pulse was used to remove the broad resonances of the protein. On-resonance and off-resonance frequencies were set at 480 Hz and 24,000 Hz, respectively. The STD NMR spectrum was obtained by a phase cycling subtraction of the on-resonance and off-resonance data acquired in an interleaved mode. The STD intensities (I_STD_) were calculated as follows:

(1) $$I_{STD}=\frac{I_{0}-I_{sat}}{I_{0}}$$

where I_0_ and I_sat_ are the intensities of a signal in the off-resonance and on-resonance spectra, respectively. The normalization of the STD intensities against the most intense signal, which is assigned a value of 100%, provides the relative STD percentages. The binding epitopes of pentasaccharides (1) and (2) upon interaction with AT were identified based on fully resolved proton signals, as illustrated in Figure 2. These resonances are delineated using a semi-quantitative color scale, where blue, orange and green dots represent relative STD intensities ranging between 100 and 80%, 79 and 60% and 59 and 40%, respectively.

As fully described in Monaco et al. (2017), the DEEP-STD NMR protocol relies on running two STD NMR experiments under two different conditions, in our case, in two solvents, and quantifying the differences between the two STD NMR data sets [17]. The result provides a differential epitope map, which is determined by calculating the DEEP-STD factor for each proton *i* (ΔSTD_i_) using the following formula:

(2) $${∆STD}_{i}=\frac{{STD}_{exp1,i}}{{STD}_{exp2,i}}-\frac{1}{n}\sum_{i}^{n}\left(\frac{{STD}_{exp1,i}}{{STD}_{exp2,i}}\right)$$

where STD_exp1,i_ and STD_exp2,i_ are the STD intensities for each proton *i* in the experiment 1 (exp1) and 2 (exp2), respectively, and *n* is the number of the ratios STD_exp1,i_/STD_exp2,i_. In this study, the experiment 1 was the experiment in D_2_O, while the experiment 2 was the experiment in H_2_O. Moreover, only the strongest DEEP-STD factors ($\left|{∆STD}_{i}\right|>0.1$) were considered significant.

For the NOESY experiment, 0.7 mg oligosaccharide was solubilized in 0.2 mL of 10 mM phosphate buffer pH 7.4 with 150 mM NaCl (D_2_O); for the tr-NOESY experiment, the sample was prepared by dissolving 0.7 mg oligosaccharide and 3.9 mg AT in 0.2 mL of the aforementioned buffer, reaching a molar ratio of ligand/protein 6:1. The final concentrations were about 2 mM for the ligand and 350 µM for the protein. All NOESY and tr-NOESY experiments were performed at 285 K. The lower temperature was used to avoid the overlapping of the H_2_O signal with the H5 signals of IdoA2S(B)/IdoA(B) and, thus, to measure the NOE and tr-NOE values for these protons. For each free induction decay (2048 × 256 points), 16 scans were collected, and the data were zero-filled to 2048 × 1024 points before the Fourier transformation. The NOESY/tr-NOESY spectra were acquired at three different mixing times (0.15 s, 0.3 s and 0.5 s). The NOE and tr-NOE values are expressed as a percentage of the mean value of the diagonal peaks of H1 GlcNS6S(E), H1 GlcNS3S6S(C), H1 IdoA2S(B)/IdoA(B) and H1 GlcNS6S(A).

### 4.3. Molecular Docking

Molecular docking was performed in Maestro using Glide (Schrodinger, LLC, New York, NY, USA) [52,53]. The crystal structure of AT was downloaded from the Protein Data Bank (PDB ID: 1AZX). The bond orders were assigned, and the hydrogens were added to all atoms in the structure. The protonation state for each residue was calculated with Epik at pH 7.4. The structure was refined to optimize the hydrogen bond network using the OPLS3e force field. The minimization was terminated when the energy gradient was lower than 10^−4^ KJ/mol·Å or the displacement (RMSD) was lower than the cut-off of 0.30 Å. The receptor grid was generated with the Receptor Grid Generation tool, setting a square box centered on the co-crystallized ligand. No constraints and no flexible side chains were included in the docking protocol. Conformations of both ligands were previously optimized using the Ligand Preparation tool. The standard precision (SP) docking mode was performed on the generated grid of the protein structure. The final evaluation of the protein–ligand binding was performed using the GlideScore and RMSD values.

### 4.4. Molecular Dynamics (MD) Simulations

The 3D models of the pentasaccharide (1)-AT and pentasaccharide (2)-AT complexes were built starting from the X-ray pentasaccharide-AT structure deposited in the Protein Data Bank (PDB ID: 1AZX). The initial conformations of pentasaccharides (1) and (2) were established by defining the interglycosidic dihedrals, φ_i_ = H1-C1-O4-C4 and ψ_i_ = C1-O4-C4-H4. For each disaccharide within the two pentasaccharides, including GlcNS6S(E)-GlcA(D), GlcA(D)-GlcNS3S6S(C), GlcNS3S6S(C)-IdoA2S(B)/IdoA(B) and IdoA2S(B)/IdoA(B)-GlcNS6S(A), their initial values were set at −49°/−14°, 59°/−6°, −59°/−26° and 57°/11°, respectively. These values were derived from the pentasaccharide co-crystallized with AT in 1AZX [39].

The topology and coordinates files were created through the AmberTools 14 package [54]. Both systems were parametrized using Amber (ff14SB) and Glycam06 forcefields for the protein and the ligand, respectively [19]. Both complexes were solvated by covering each of them with a 15 Å layer of water molecules (TIP3P) to obtain an orthogonal bounding box of hedges approximately 100 Å long. No counterions were added to the system. The equilibration protocol and production dynamics were performed with the sander and pmemd modules of the Amber package, respectively. After the minimization of the solvent (two steps of minimization: 500 steepest descent followed by 500 conjugate gradient cycles; harmonic force constant at 500 Kcal/mol^·^Å^2^), the entire system was minimized (two steps of minimization: 1000 steepest descent followed by 1500 conjugate gradient cycles). Before equilibrating at constant pressure (1 atm), each system was heated to 300 K at constant volume (50 ps MD simulation). The cell density was then equilibrated (100 ps MD simulation), sampling the NPT ensemble. The pentasaccharide (1)-AT and pentasaccharide (2)-AT complexes were simulated for 1 µs, sampling the NPT ensemble without any restraint. The first 0.5 µs were considered as the equilibration stage, while the last 0.5 µs were defined as the production stage. The conformational equilibration stage was established by plotting vs. time the RMSD of the ligand in comparison to its initial position (time 0) and the interglycosidic dihedral angles, φ_i_(t)/ψ_i_(t), until a stationary oscillatory behavior was observed for all variables (Figures S7, S24 and S25). In all cases, periodic boundary conditions and the particle mesh Ewald method were applied. A cut-off of 10 Å was used for all non-bonded interactions. A Langevin thermostat with a collision frequency of 3 ps^−1^ and a Berendsen barostat with a relaxation time of 2 ps were used. The SHAKE algorithm was employed to restrain all bonds involving hydrogen, allowing a timestep of 2 fs.

The RMSD, RMSF and clustering analyses were carried out with CPPTRAJ [55] included in Ambertool 14 package [54]. Clustering of trajectories was performed using the DBSCAN algorithm with the minimum number of points allowed for the cluster set to 25 and epsilon set to 0.5 Å [56].

The Ramachandran plots and the density color maps were generated using the statistical software R 4.3.1 (R Core Team, 2013) and the hexbin package [57,58].

The Poisson Boltzmann free energy of binding (${∆G}_{PB}^{bind}$) was estimated applying the molecular mechanics Poisson Boltzmann surface area (MMPBSA) method (MMPBSA.py application) [59,60]. For each protein–ligand complex, the ${∆G}_{PB}^{bind}$ was determined as average on the production stage of the MD simulation (between 0.5 and 1 µs) with a sampling frequency of 2 ns and a sample size of 5000 poses covering a range of 500 ns. The standard error of the mean (SEM) of ${∆G}_{PB}^{bind}$ was calculated as σ/√N, where *σ* represents the estimated standard deviation and *N* indicates the number of samples.

The profile of the local concentration of the water molecules surrounding selected atoms (or a set of atoms) of protein residues, ligand units or protons was investigated using the radial distribution function [g(r)]:

(3) $$g(r)=\underset{\mathrm{dr}\to 0}{\lim}\left[\frac{V}{{4\pi N}_{pair}}\cdot \frac{p(r)}{r^{2}dr}\right]$$

where the variable *r* represents the distance between the reference atom (or set of atoms) and the oxygen atom of each water molecule, *g*(*r*) *dr* reflects the concentration of water molecules in a spherical layer of infinitesimal thickness (between *r* and *r + dr*) centered on the reference atom, *V* is the volume of the simulation cell, *Npair* denotes the number of possible atom pairs [61,62,63,64].

### 4.5. Model Validation by RedMat

The RedMat analysis was employed to calculate the theoretical binding epitope from the MD trajectories of the pentasaccharide (1)-AT and pentasaccharide (2)-AT complexes [42]. For the RedMat calculation, the following parameters were set: the NMR spectrometer frequency at 600 MHz, the complex rotational correlation time at 34.956 ns, the concentrations of ligand and protein at 2000 μM and 50 μM, respectively, and the cut-off distance at 10 Å. The agreement between the theoretical and experimental binding epitopes was evaluated using the R-factor (R-NOE):

(4) $$R\text{-}NOE=\sqrt{\frac{\sum {\left({STD}^{exp,k}-{STD}^{calc,k}\right)}^{2}}{\sum {\left({STD}^{exp,k}\right)}^{2}}}$$

where STD^exp,k^ is the experimental STD value for a proton *k* and STD^calc,k^ is the STD value simulated using the RedMat algorithm. The experimental binding epitope was determined by employing the smallest saturation time still producing integrable signals (0.5 s), so that the experimental STD values could be considered not significantly affected by differential proton relaxation rates without the need of determining STD intensities at the saturation time of 0 (STD_0_).

## Figures and Tables

**Table 1:** The most populated states of each dihedral angle φ_i_/ψ_i_ of pentasaccharides (1) and (2) in the AT-bound state. The relative population percentages (%) for the glycosidic linkages characterized by more than one conformational state are reported in brackets.

System	φ_1_/ψ_1_	φ_2_/ψ_2_	φ_3_/ψ_3_	φ_4_/ψ_4_
Pentasaccharide (1)-AT	−54°/−32°	50°/9°	−61°/−46°	59°/12°
Pentasaccharide (2)-AT	−41°/−29°	43°/17°	−45°/−42°	44°/6° (78%); −37°/−28° (22%)

**Table 2:** Selected contact distances in the pentasaccharide-AT (PDB ID: 1AZX), pentasaccharide (1)-AT and pentasaccharide (2)-AT complexes. The average distances between key interacting groups were derived from the last 500 ns of MD simulations. Distances (Å) were measured using specific atoms: sulfur for sulfate groups, carbon for carboxylic groups, nitrogen for lysine side chains and carbon for guanidinium groups of arginine.

	Pentasaccharide-AT (PDB ID: 1AZX)	Pentasaccharide (1)-AT	Pentasaccharide (2)-AT
Contacts	Dist.	Avg. Dist.	Avg. Dist.
GlcNS6S(E)(6S)-K125	6.0	4.9	6.1
GlcNS6S(E)(6S)-R129	4.9	4.5	5.4
GlcA(D)(COO^−^)-K125	4.9	7.3	4.5
GlcNS3S6S(C)(NS)-R13	4.5	5.6	4.8
GlcNS3S6S(C)(NS)-K114	3.8	5.1	4.1
GlcNS3S6S(C)(6S)-R13	10.9	12.2	5.1
GlcNS3S6S(C)(3S)-K114	3.1	3.9	4.9
GlcNS3S6S(C)(6S)-R46	9.7	8.1	14.7
IdoA2S(B)(2S)/ IdoA(B)(2OH)-R13	5.7	6.8	7.0
IdoA2S(B)(COO^−^)/ IdoA(B)(COO^−^)-R46	5.1	5.5	10.6
IdoA2S(B)(COO^−^) /IdoA(B)(COO^−^)-R47	5.0	4.4	6.8
IdoA2S(B)(COO^−^)/ IdoA(B)(COO^−^)-K114	3.9	3.7	6.6
GlcNS6S(A)(NS)-R46	6.8	7.4	11.4
GlcNS6S(A)(NS)-R47	4.3	4.3	8.6
GlcNS6S(A)(6S)-R13	4.7	7.3	11.2
GlcNS6S(A)(6S)-K114	5.1	5.8	5.7

## Data Availability

Data supporting the results of the study are available from the corresponding authors upon request.

## Supplementary Materials

The following supporting information can be downloaded at https://www.mdpi.com/article/10.3390/ijms25094669/s1.
