# Supplementary material for: Differential Solvent DEEP-STD NMR and MD Simulations Enable the Determinants of the Molecular Recognition of Heparin Oligosaccharides by Antithrombin to Be Disentangled
Source: Int J Mol Sci. 2024 Apr 25;25(9):4669. doi: 10.3390/ijms25094669 (PMC11083112; doi:10.3390/ijms25094669)
Supplement: Supplementary file 1 [file ijms-25-04669-s001.zip › ijms-2916056-supplementary.pdf]

Differential solvent DEEP-STD NMR and MD simulations enable the determinants of the molecular recognition of heparin oligosaccharides by antithrombin to be disentangled

Michela Parafioriti [1], Stefano Elli [1], Juan C. Muñoz-García [2], Jonathan Ramírez-Cárdenas [2], Edwin A. Yates [3,4], Jesús Angulo [2,*] and Marco Guerrini [1,*]

[1] Istituto di Ricerche Chimiche e Biochimiche "G. Ronzoni", Via Giuseppe Colombo 81, 20133, Milano, Italia
[2] Instituto de Investigationes Químicas (IIQ)-Consejo Superior de Investigaciones Científicas (CSIC), Avenida Americo Vespucio 49, 41092, Sevilla, España
[3] Department of Biochemistry and Systems Biology, Institute of Systems, Molecular and Integrative Biology, University of Liverpool, L69 7ZB Liverpool, United Kingdom
[4] Centre for Glycoscience, Keele University, Newcastle-Under-Lyme, ST5 5BG Staffordshire, United Kingdom
* Correspondence: J.A. j.angulo@iiq.csic.es; M.G. guerrini@ronzoni.it

Supporting Information

Index

Figure S1. STD NMR analysis of pentasaccharide (1) in interaction with AT conducted in D_2O buffer.

Figure S2. STD NMR analysis of pentasaccharide (1) in interaction with AT conducted in H_2O buffer.

Figure S3. STD NMR analysis of pentasaccharide (2) in interaction with AT conducted in D_2O buffer.

Figure S4. STD NMR analysis of pentasaccharide (2) in interaction with AT conducted in H_2O buffer.

Table S1. Absolute and relative STD percentages (STD %) of selected protons of pentasaccharide (1).

Table S2. Absolute and relative STD percentages (STD %) of selected protons of pentasaccharide (2).

Table S3. DEEP-STD NMR analysis using different solvents (D_2O/H_2O) of the pentasaccharide (1)-AT and pentasaccharide (2)-AT complexes.

Table S4. $^3J_{H-H}$ coupling constants (Hz) of the sugar units of pentasaccharide (1) in the unbound state.

Table S5. $^3J_{H-H}$ coupling constants (Hz) of the sugar units of pentasaccharide (2) in the unbound state.

Figure S5. NOESY spectrum of pentasaccharide (1) [panel (a)]. Tr-NOESY spectrum of pentasaccharide (1) interacting with AT [panel (b)].

Figure S6. NOESY spectrum of pentasaccharide (2) [panel (a)]. Tr-NOESY spectrum of pentasaccharide (2) interacting with AT [panel (b)].

Table S6. H1-H2 NOEs and tr-NOEs of GlcNS6S(E, C, A) of pentasaccharide (1) in the unbound and AT-bound states.

Table S7. H1-H2 NOEs and tr-NOEs of GlcNS6S(E, C, A) of pentasaccharide (2) in the unbound and AT-bound states.

Table S8. H5-H2 and H5-H4 NOEs and tr-NOEs of IdoA(B) of pentasaccharide (2) in the unbound and AT-bound states.

Table S9. H1-H6 and H1-H4 interglycosidic NOEs and tr-NOEs characterizing the backbone conformation of pentasaccharide (1) in the unbound and AT-bound states.

Table S10. H1-H6 (or H1-H3) and H1-H4 interglycosidic NOEs and tr-NOEs characterizing the backbone conformation of pentasaccharide (2) in the unbound and AT-bound states.

Figure S7. RMSD analysis of pentasaccharides (1) and (2) in complex with AT.

Figure S8. RMSF analysis of the ligand units and key protein residues in the pentasaccharide (1)-AT and pentasaccharide (2)-AT complexes.

Figure S9. Conformation of IdoA2S(B) and IdoA(B).

Figure S10. Structures of pentasaccharides (1) and (2) in the AT-bound state selected from the MD trajectories considering the most populated state for each dihedral angle.

Figure S11. Conformers of pentasaccharides (1) and (2) in the AT-bound state obtained by clustering analysis.

Table S11. Dihedral angles (φ_i/ψ_i) of the clustered conformers of pentasaccharides (1) and (2) in the AT-bound state.

Figure S12. Distances (Å) of GlcNS6S(E)(6S) to K125 and R129 versus MD simulation time (ns) in the pentasaccharide (1)-AT and pentasaccharide (2)-AT complexes.

Figure S13. Distance (Å) between GlcA(D)(COO^-) and K125 versus MD simulation time (ns) in the pentasaccharide (1)-AT and pentasaccharide (2)-AT complexes.

Figure S14. Distances (Å) of GlcNS3S6S(C)(NS) to R13 and K114, GlcNS3S6S(C)(3S) to K114, GlcNS3S6S(C)(6S) to R46 versus MD simulation time (ns) in the pentasaccharide (1)-AT and pentasaccharide (2)-AT complexes.

Figure S15. Distances (Å) of IdoA2S(2S)/IdoA(B)(2OH) to R13, IdoA2S/IdoA(B)(COO^-) to R46, R47 and K114 versus MD simulation time (ns) in the pentasaccharide (1)-AT and pentasaccharide (2)-AT complexes.

Figure S16. Distances (Å) of GlcNS6S(A)(NS) to R46 and R47, GlcNS6S(A)(6S) to R13 and K114 versus MD simulation time (ns) in the pentasaccharide (1)-AT and pentasaccharide (2)-AT complexes.

Figure S17. Radial distribution function [g(r)] curves between selected protein residues and water molecules in the pentasaccharide (1)-AT and pentasaccharide (2)-AT complexes.

Figure S18. Radial distribution function [g(r)] curves between ligand units and water molecules in the pentasaccharide (1)-AT and pentasaccharide (2)-AT complexes.

Figure S19. Radial distribution function [g(r)] curves between selected ligand protons and water molecules in the pentasaccharide (1)-AT and pentasaccharide (2)-AT complexes.

Table S12. Comparison between the binding epitopes obtained experimentally (Exp STD) and calculated during MD simulation (Sim STD) of pentasaccharide (1) in complex with AT.

Table S13. Comparison between the binding epitopes obtained experimentally (Exp STD) and calculated during MD simulation (Sim STD) of pentasaccharide (2) in complex with AT.

Figure S20. Evolution of the R-factor (R-NOE) of pentasaccharide (1) over the last 500 ns of MD simulation.

Figure S21. Evolution of the R-factor (R-NOE) of pentasaccharide (2) over the last 500 ns of MD simulation.

Table S14. $^{1}H/^{13}C$ chemical shift assignment of pentasaccharide (1).

Table S15. $^{1}H/^{13}C$ chemical shift assignment of pentasaccharide (2).

Figure S22. ^{1}H-^{13}C HSQC NMR spectrum of pentasaccharide (1).

Figure S23. ^{1}H-^{13}C HSQC NMR spectrum of pentasaccharide (2).

Figure S24. Conformations of the φ_i/ψ_i dihedral angles of pentasaccharide (1) in complex with AT.

Figure S25. Conformations of the φ_i/ψ_i dihedral angles of pentasaccharide (2) in complex with AT.

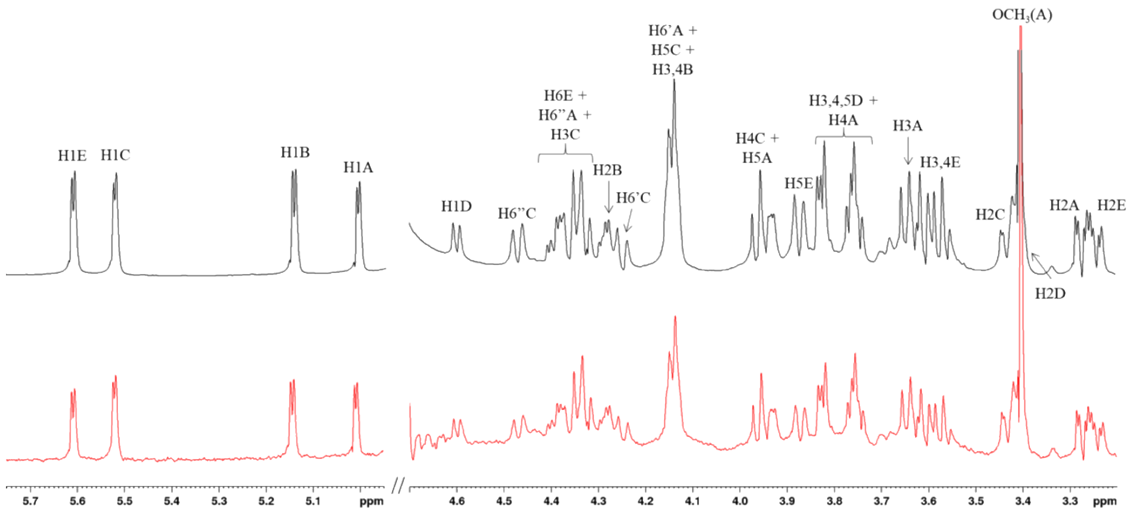

Figure S1. STD NMR analysis of pentasaccharide (1) in interaction with AT conducted in D_2O buffer. Superimposition of the STD NMR spectrum (red line) and the reference spectrum (black line) of the pentasaccharide (1)-AT mixture in D_2O.

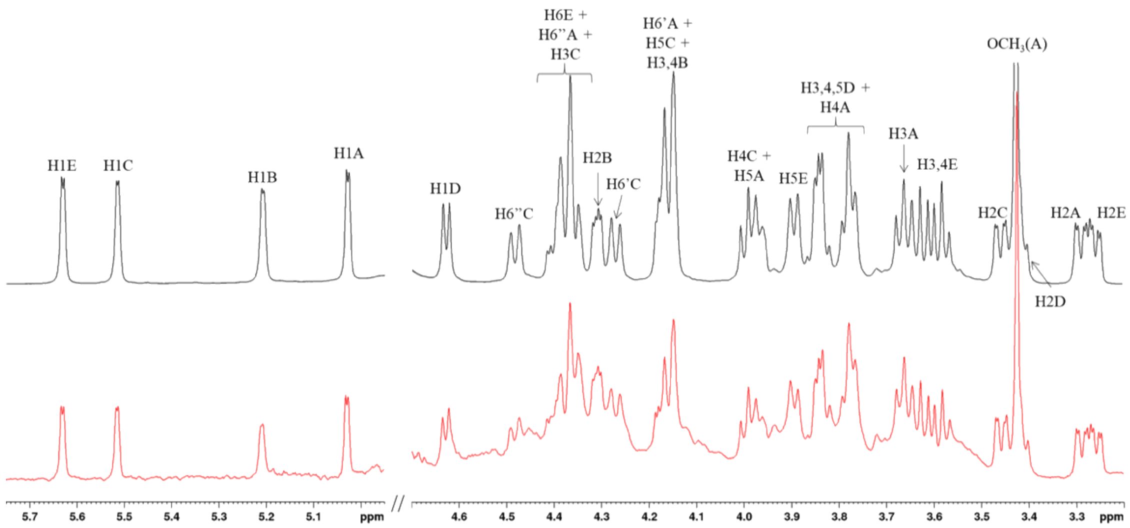

Figure S2. STD NMR analysis of pentasaccharide (1) in interaction with AT conducted in H_2O buffer. Superimposition of the STD NMR spectrum (red line) and the reference spectrum (black line) of the pentasaccharide (1)-AT mixture in H_2O.

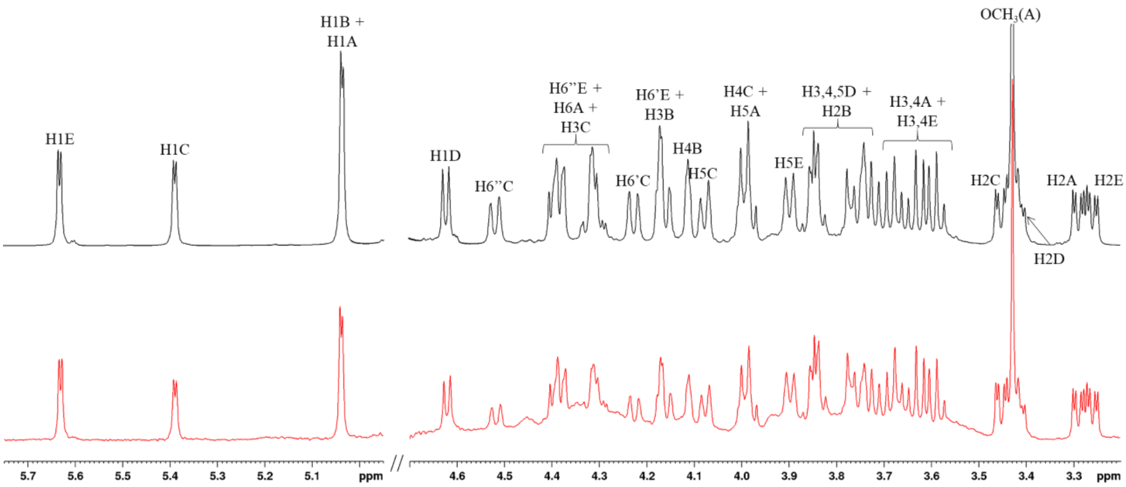

Figure S3. STD NMR analysis of pentasaccharide (2) in interaction with AT conducted in D_2O buffer. Superimposition of the STD NMR spectrum (red line) and the reference spectrum (black line) of the pentasaccharide (2)-AT mixture in D_2O.

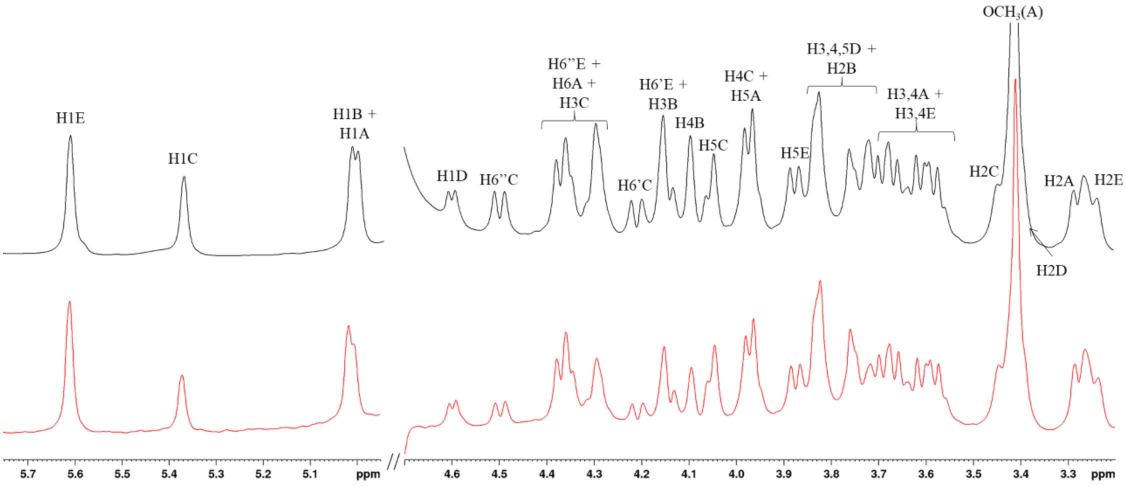

Figure S4. STD NMR analysis of pentasaccharide (2) in interaction with AT conducted in H_2O buffer. Superimposition of the STD NMR spectrum (red line) and the reference spectrum (black line) of the pentasaccharide (2)-AT mixture in H_2O.

Table S1. Absolute and relative STD percentages (STD %) of selected protons of pentasaccharide (1).

	Pentasaccharide (1)-AT			
	**Absolute STD %**		**Relative STD %**	
**Proton**	**D_2O**	**H_2O**	**D_2O**	**H_2O**
H1E	1.04	1.46	70	69
H2E	1.16	1.52	78	72
H5E	1.03	1.27	69	60
H1D	1.07	1.29	72	61
H1C	1.04	1.49	70	70
H2C	1.49	2.11	100	100
H6′′C	1.22	1.58	82	75
H1B	0.95	1.37	64	65
H2B	1.21	1.42	81	67
H1A	0.98	1.38	66	66
H2A	1.42	1.65	95	78
H3A	1.31	1.57	88	74
OCH_3(A)	1.22	1.63	82	77
	Sum	***Sum***		
	15.16	*19.73*		

Table S2. Absolute and relative STD percentages (STD %) of selected protons of pentasaccharide (2).

	Pentasaccharide (2)-AT			
	**Absolute STD %**		**Relative STD %**	
**Proton**	**D_2O**	**H_2O**	**D_2O**	**H_2O**
H1E	2.01	2.10	76	90
H2E	2.14	1.59	81	68
H5E	1.93	1.59	73	68
H1D	1.89	1.27	72	54
H1C	1.72	1.35	65	58
H2C	2.44	2.15	93	92
H6′′C	1.74	1.32	66	57
H4B	1.45	1.00	55	43
H2A	2.63	2.34	100	100
OCH_3(A)	1.56	1.22	59	52
	Sum	***Sum***		
	19.50	*15.94*		

Table S3. DEEP-STD NMR analysis using different solvents (D_2O/H_2O) of the pentasaccharide (1)-AT and pentasaccharide (2)-AT complexes.

	Pentasaccharide (1)-AT				*Pentasaccharide (2)-AT*			
**Proton**	**STD % D_2O**	**STD % H_2O**	**STD D_2O/H_2O Ratio**	**ΔSTD**	**STD % D_2O**	**STD % H_2O**	**STD D_2O/H_2O Ratio**	**ΔSTD**
H1E	1.04	1.46	0.71	–0.05	2.01	2.10	0.96	–0.28
H2E	1.16	1.52	0.76	0.00	2.14	1.59	1.34	0.11
H5E	1.03	1.27	0.82	0.05	1.93	1.59	1.21	–0.02
H1D	1.07	1.29	0.83	0.06	1.89	1.27	1.48	0.25
H1C	1.04	1.49	0.70	–0.07	1.72	1.35	1.28	0.04
H2C	1.49	2.11	0.71	–0.06	2.44	2.15	1.14	–0.10
H6″C	1.22	1.58	0.78	0.01	1.74	1.32	1.31	0.08
H2A	1.42	1.65	0.86	0.09	2.63	2.34	1.12	–0.11
OCH_3(A)	1.22	1.63	0.75	–0.02	1.56	1.22	1.28	0.04
	**Sum**	**Sum**	**Average**		**Sum**	**Sum**	**Average**	
	10.71	13.99	0.77		18.05	14.94	1.24	
			**St. Dev.**				**St. Dev.**	
			0.06				0.15	

Table S4. $^3J_{H\text{-}H}$ coupling constants (Hz) of the sugar units of pentasaccharide (1) in the unbound state.

$^3J_{H\text{-}H}$	GlcNS6S(E)	GlcA(D)	GlcNS3S6S(C)	IdoA2S(B)	GlcNS6S(A)
$^3J_{1\text{-}2}$	3.7	7.9	3.5	3.7	3.6
$^3J_{2\text{-}3}$	10.0	9.9	10.7	7.3	10.5
$^3J_{3\text{-}4}$	-	-	-	-	-
$^3J_{4\text{-}5}$	-	-	-	3.1	-

Table S5. $^3J_{H\text{-}H}$ coupling constants (Hz) of the sugar units of pentasaccharide (2) in the unbound state.

$^3J_{H\text{-}H}$	GlcNS6S(E)	GlcA(D)	GlcNS3S6S(C)	IdoA(B)	GlcNS6S(A)
$^3J_{1\text{-}2}$	3.7	7.9	3.5	-	3.5
$^3J_{2\text{-}3}$	9.9	9.3	10.6	-	10.6
$^3J_{3\text{-}4}$	-	-	-	-	-
$^3J_{4\text{-}5}$	-	-	-	2.5	-

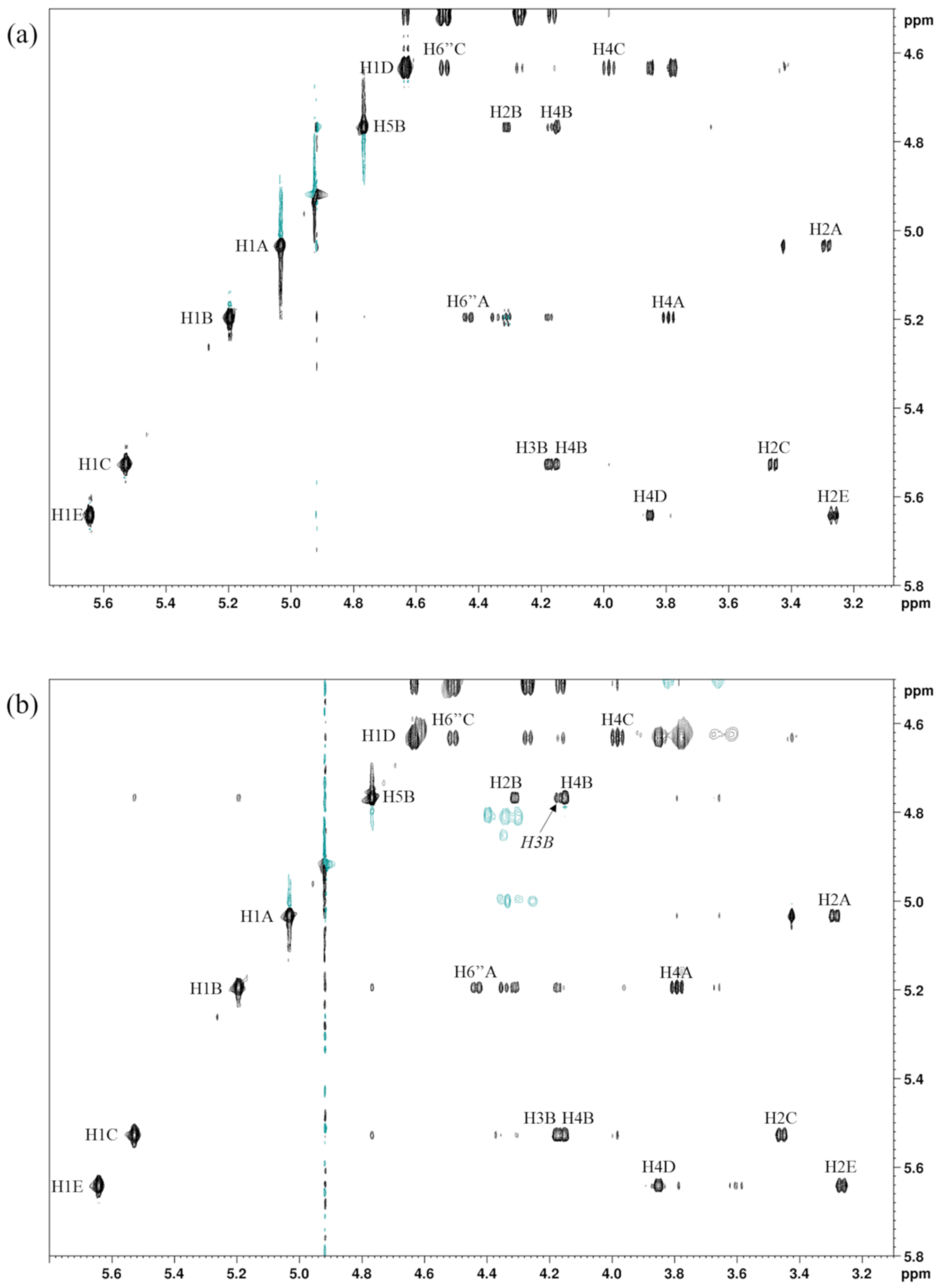

Figure S5. NOESY spectrum of pentasaccharide (1) [panel (a)]. Tr-NOESY spectrum of pentasaccharide (1) interacting with AT [panel (b)].

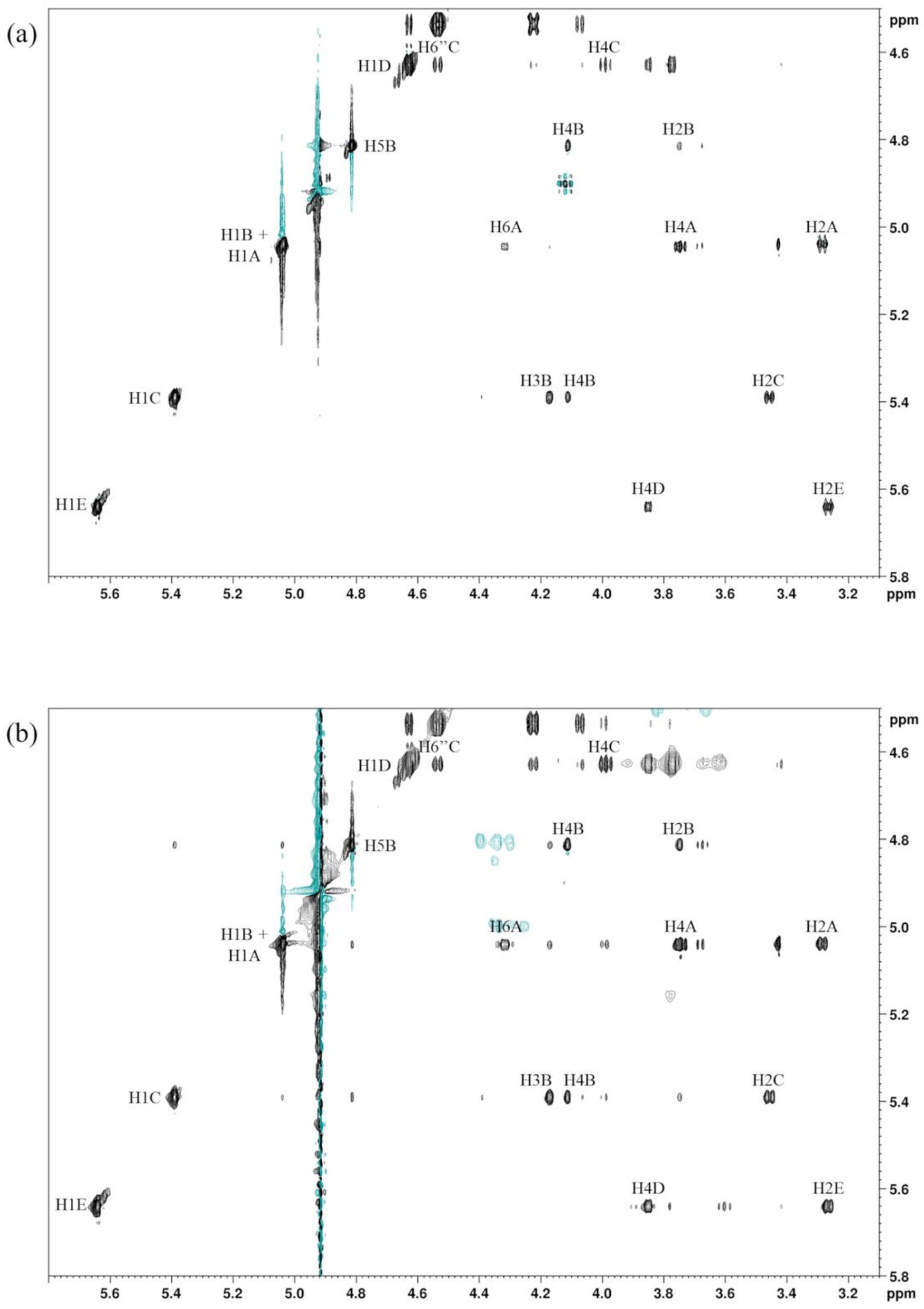

Figure S6. NOESY spectrum of pentasaccharide (2) [panel (a)]. Tr-NOESY spectrum of pentasaccharide (2) interacting with AT [panel (b)].

Table S6. H1-H2 NOEs and tr-NOEs of GlcNS6S(E, C, A) of pentasaccharide (1) in the unbound and AT-bound states. The mixing time (T_{mix}) is reported in seconds. The NOE and tr-NOE intensities are in percentage.

	GlcNS6S(E)		*GlcNS3S6S(C)*		*GlcNS6S(A)*	
**T_{mix}**	**NOE**	**tr-NOE**	**NOE**	**tr-NOE**	**NOE**	**tr-NOE**
	H1-H2	H1-H2	H1-H2	H1-H2	H1-H2	H1-H2
0.15	4.1	9.4	4.4	9.6	4.4	8.4
0.3	8.7	20.6	8.6	19.2	6.8	18.1
0.5	14.3	35.2	14.0	31.3	12.1	31.6

Table S7. H1-H2 NOEs and tr-NOEs of GlcNS6S(E, C, A) of pentasaccharide (2) in the unbound and AT-bound states. The mixing time (T_{mix}) is reported in seconds. The NOE and tr-NOE intensities are in percentage.

	GlcNS6S(E)		*GlcNS3S6S(C)*		*GlcNS6S(A)*	
**T_{mix}**	**NOE**	**tr-NOE**	**NOE**	**tr-NOE**	**NOE**	**tr-NOE**
	H1-H2	H1-H2	H1-H2	H1-H2	H1-H2	H1-H2
0.15	4.1	9.4	5.3	8.8	4.2	8.4
0.3	8.7	20.2	8.0	17.8	7.3	18.2
0.5	14.3	34.9	13.1	27.4	11.6	33.1

Table S8. H5-H2 and H5-H4 NOEs and tr-NOEs of IdoA(B) of pentasaccharide (2) in the unbound and AT-bound states. The mixing time (T_{mix}) is reported in seconds. The NOE and tr-NOE intensities are in percentage. The ratio H5-H2/H5-H4 is shown in brackets.

	IdoA(B)	
**T_{mix}**	**NOE**	**tr-NOE**
	H5-H2/ H5-H4	H5-H2/ H5-H4
0.15	0.8/3.2 (0.3)	5.1/7.1 (0.7)
0.3	1.7/6.5 (0.3)	10.5/15.4 (0.7)
0.5	3.2/11.0 (0.3)	16.9/25.9 (0.7)

Table S9. H1-H6 and H1-H4 interglycosidic NOEs and tr-NOEs characterizing the backbone conformation of pentasaccharide (1) in the unbound and AT-bound states. The mixing time (T_{mix}) is reported in seconds. The NOE and tr-NOE intensities are in percentage. The ratios H1-H6/H1-H4 are shown in brackets.

	GlcNS6S(E)-GlcA(D)		*GlcA(D)-GlcNS3S6S(C)*		*IdoA2S(B)-GlcNS6S(A)*	
**T_{mix}**	**NOE**	**tr-NOE**	**NOE**	**tr-NOE**	**NOE**	**tr-NOE**
	H1-H4	H1-H4	H1-H6″/ H1-H4	H1-H6″/ H1-H4	H1-H6/ H1-H4	H1-H6/ H1-H4
0.15	3.0	8.2	4.6/2.9 (1.6)	9.4/8.5 (1.1)	2.3/3.1 (0.7)	5.1/10.2 (0.5)
0.3	6.3	18.2	7.5/5.5 (1.4)	12.6/19.4 (0.6)	4.3/6.3 (0.7)	9.6/18.2 (0.5)
0.5	10.8	30.9	9.9/8.6 (1.2)	14.8/28.4 (0.5)	6.5/10.2 (0.6)	15.5/27.8 (0.6)

Table S10. H1-H6 (or H1-H3) and H1-H4 interglycosidic NOEs and tr-NOEs characterizing the backbone conformation of pentasaccharide (2) in the unbound and AT-bound states. The mixing time (T_{mix}) is reported in seconds. The NOE and tr-NOE intensities are in percentage. The ratios H1-H6/H1-H4 and H1-H3/H1-H4 are shown in brackets.

	GlcNS6S(E)-GlcA(D)		*GlcA(D)-GlcNS3S6S(C)*		*GlcNS3S6S(C)-IdoA(B)*		*IdoA(B)-GlcNS6S(A)*	
**T_{mix}**	**NOE**	**tr-NOE**	**NOE**	**tr-NOE**	**NOE**	**tr-NOE**	**NOE**	**tr-NOE**
	H1-H4	H1-H4	H1-H6''/ H1-H4	H1-H6''/ H1-H4	H1-H3/ H1-H4	H1-H3/ H1-H4	H1-H6/ H1-H4	H1-H6/ H1-H4
0.15	3.1	8.6	4.1/2.8 (1.4)	9.1/8.1 (1.1)	5.1/2.4 (2.2)	10.8/5.2 (2.1)	1.8/5.2 (0.3)	4.5/13.1 (0.3)
0.3	6.4	18.7	7.3/5.4 (1.3)	11.8/18.4 (0.6)	10.1/4.9 (2.1)	20.7/10.5 (2.0)	3.6/10.5 (0.3)	11.5/27.5 (0.4)
0.5	10.9	33.0	8.9/8.1 (1.1)	14.5/25.8 (0.6)	16.5/8.2 (2.0)	32.7/17.7 (1.8)	6.1/17.8 (0.3)	20.8/45.4 (0.5)

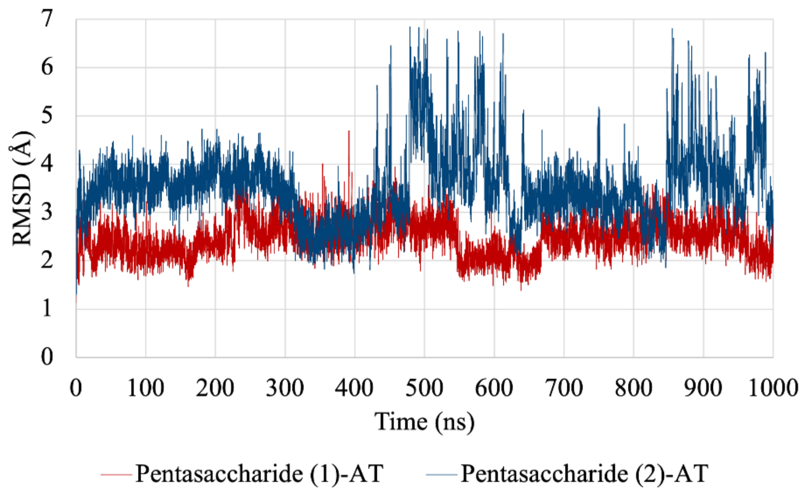

Figure S7. RMSD analysis of pentasaccharides (1) and (2) in complex with AT. The red and blue lines show the RMSD calculated for pentasaccharides (1) and (2) in the AT-bound state during the equilibration and production stages of MD simulations. All distances are expressed in Ångström (Å).

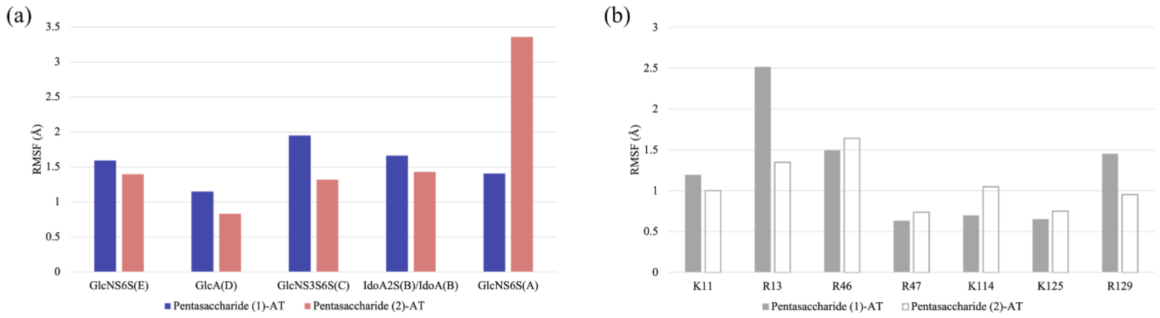

Figure S8. RMSF analysis of the ligand units and key protein residues in the pentasaccharide (1)-AT and pentasaccharide (2)-AT complexes. Panel (a): RMSFs are defined for each ligand units of both pentasaccharides (1) and (2) in the AT-bound state during the last 500 ns MD simulations. Panel (b): RMSFs are delineated for the protein residues in the AT binding site of the pentasaccharide (1)-AT and pentasaccharide (2)-AT complexes during the last 500 ns MD simulations. All distances are expressed in Ångström (Å).

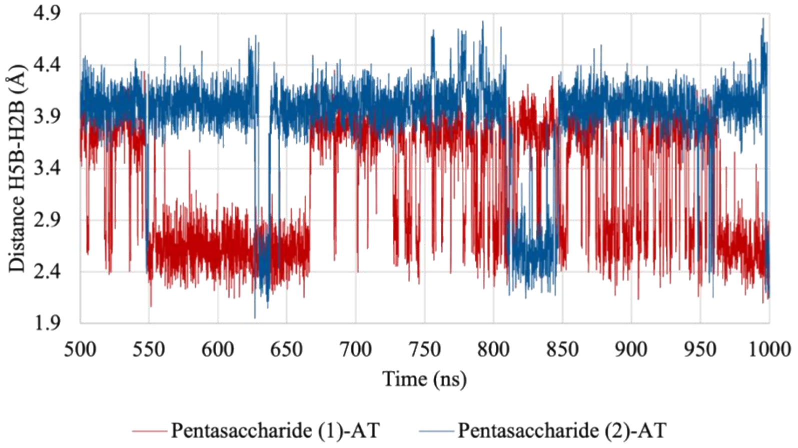

Figure S9. Conformation of IdoA2S(B) and IdoA(B). Plot of the H5B-H2B distance (Å) in the pentasaccharide (1)-AT and pentasaccharide (2)-AT complexes during the last 500 ns MD simulations.

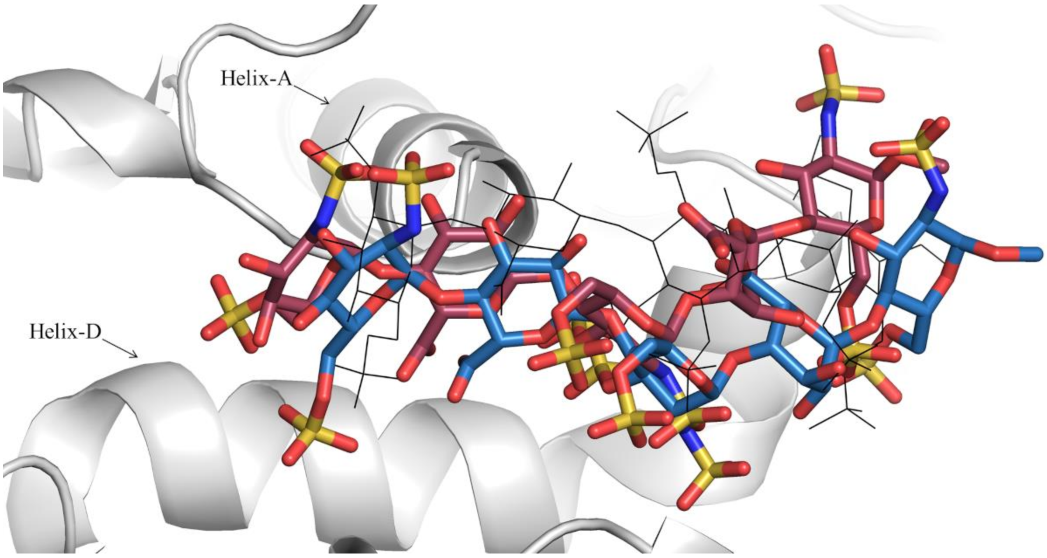

Figure S10. Structures of pentasaccharides (1) and (2) in the AT-bound state selected from the MD trajectories considering the most populated states for each dihedral angle. Pentasaccharide (1) presents a single geometry for each dihedral angle φ_i/ψ_i, while pentasaccharide (2) exhibits two conformational states for the dihedral angle φ_4/ψ_4. Pentasaccharide (1) is displayed as black lines; pentasaccharide (2) with the dihedral angle φ_4/ψ_4 of 44°/6° is drawn as fuchsia, red, blue and yellow tubes corresponding to carbon, oxygen, nitrogen and sulfur atoms; pentasaccharide (2) with the dihedral angle φ_4/ψ_4 of –37°/–28° is reported as light blue, red, blue and yellow tubes indicating carbon, oxygen, nitrogen and sulfur atoms; AT is shown as gray ribbon.

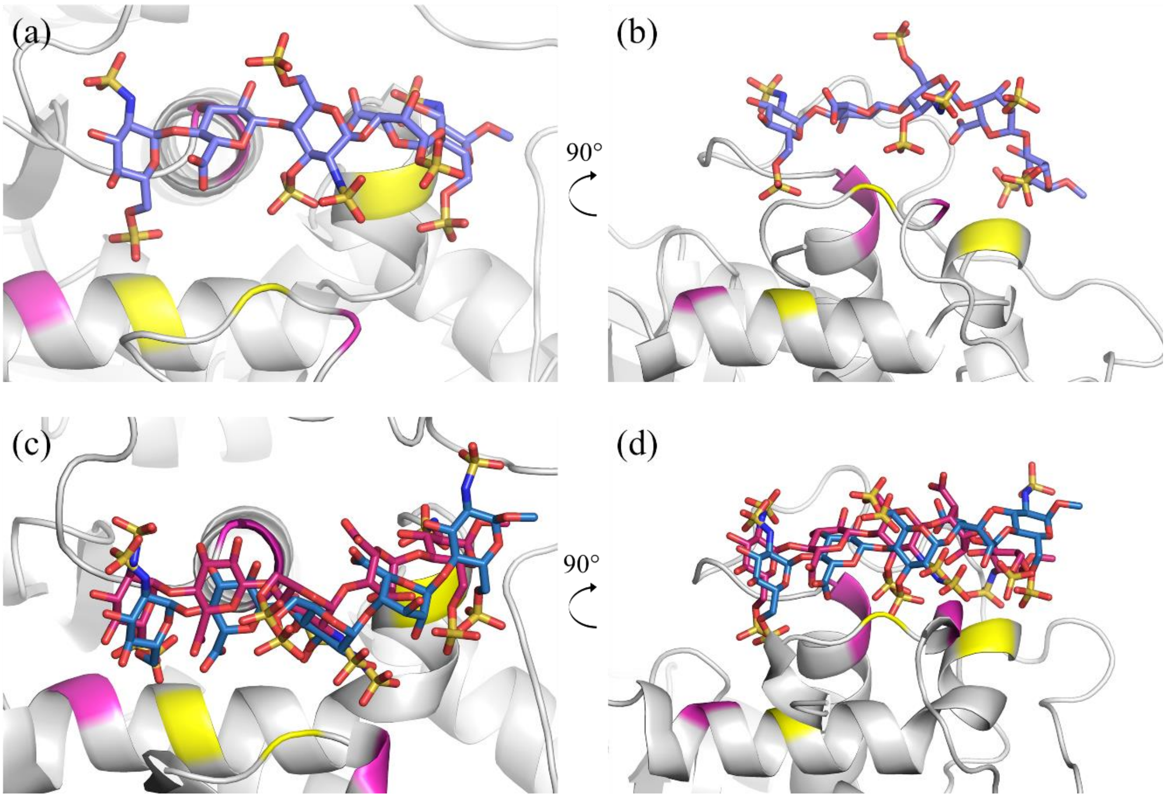

Figure S11. Conformers of pentasaccharides (1) and (2) in the AT-bound state obtained by clustering analysis. For pentasaccharide (1), a single cluster was found [panels (a) and (b)], while for pentasaccharide (2), two dominant clusters were collected [panels (c) and (d)]. Pentasaccharide (1) is displayed as purple, red, blue and yellow tubes corresponding to carbon, oxygen, nitrogen and sulfur atoms; pentasaccharide (2) is drawn as light blue (conformer 1 in Table S11) or fuchsia (conformer 2 in Table S11), red, blue and yellow tubes indicating carbon, oxygen, nitrogen and sulfur atoms; AT is shown as gray ribbon.

Table S11. Dihedral angles (φ_i/ψ_i) of the clustered conformers of pentasaccharides (1) and (2) in the AT-bound state.

System	Conformer	φ_1/ψ_1	φ_2/ψ_2	φ_3/ψ_3	φ_4/ψ_4
Pentasaccharide (1)-AT	1 (93%)	-46°/-35°	43°/-3°	-67°/-44°	56°/12°
Pentasaccharide (2)-AT	1 (33%)	-27°/-29°	39°/10°	-57°/-44°	36°/10°
Pentasaccharide (2)-AT	2 (8%)	-36°/-37°	51°/17°	-17°/-35°	-36°/-24°

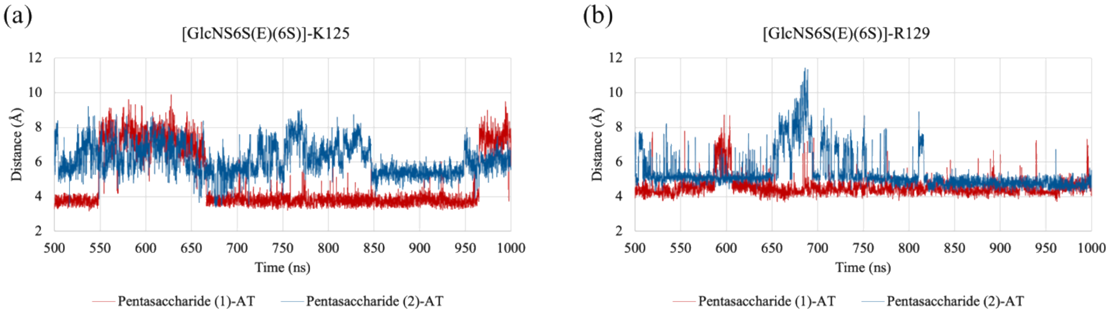

Figure S12. (a) Distances of GlcNS6S(E)(6S) to K125 versus MD simulation time in the pentasaccharide (1)-AT (red line) and pentasaccharide (2)-AT (blue line) complexes. (b) Distances of

GlcNS6S(E)(6S) to R129 versus MD simulation time in the pentasaccharide (1)-AT (red line) and pentasaccharide (2)-AT (blue line) complexes. Distances are in angstrom (Å), time is in nanoseconds (ns)

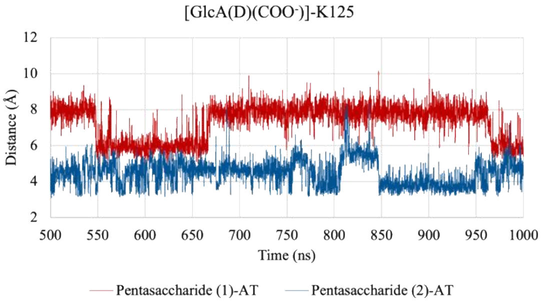

Figure S13. Distance between the carboxyl group of GlcA(D) and the NH_3^+ group of K125 versus MD simulation time in the pentasaccharide (1)-AT (red line) and pentasaccharide (2)-AT (blue line) complexes. Distances are in angstrom (Å), time is in nanoseconds (ns).

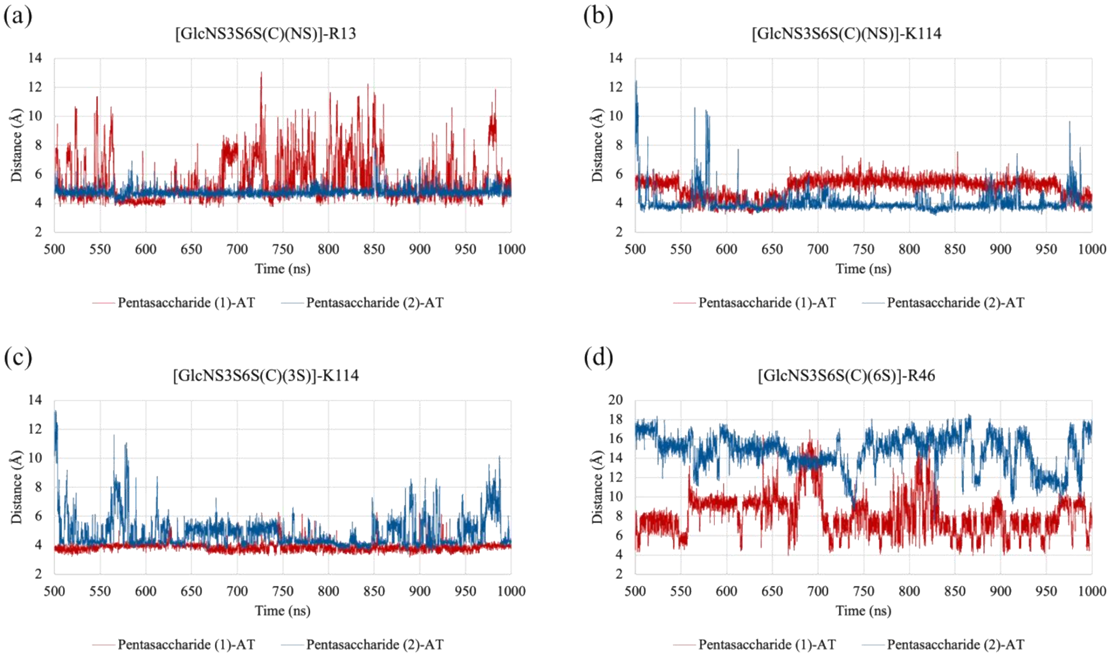

Figure S14. Distances of GlcNS3S6S(C)(NS) to R13 (a panel) and K114 (b panel), GlcNS3S6S(C)(3S) to K114 (c panel), GlcNS3S6S(C)(6S) to R46 (d panel) versus MD simulation time in the pentasaccharide (1)-AT (red line) and pentasaccharide (2)-AT (blue line) complexes. Distances are in angstrom (Å), time is in nanoseconds (ns).

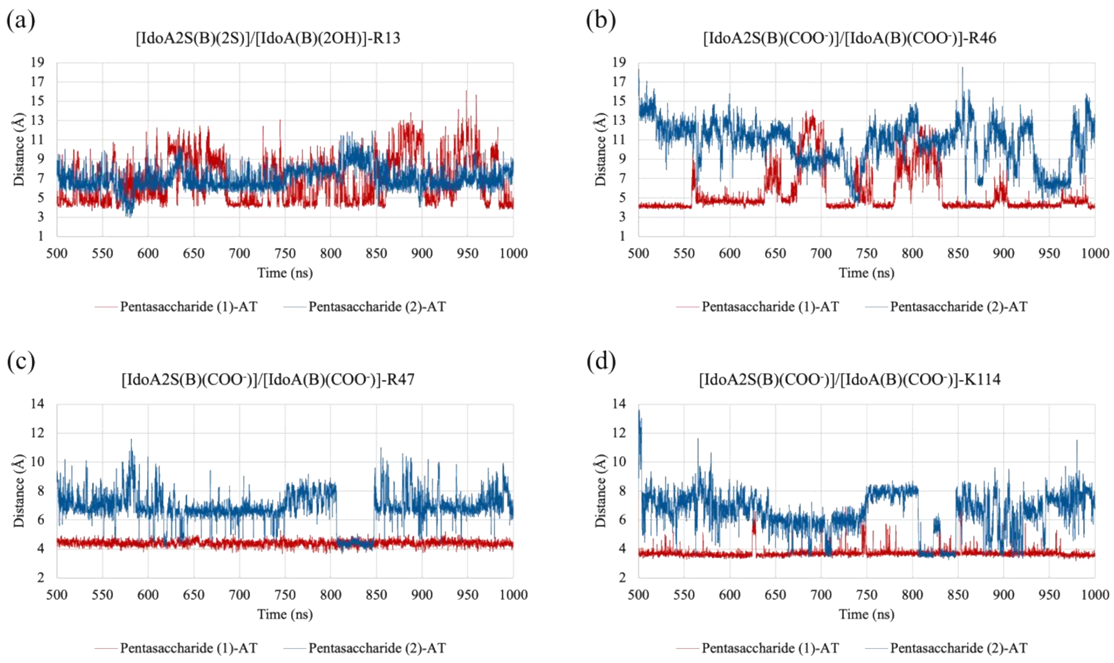

Figure S15. Distances of IdoA2S(2S)/IdoA(B)(2OH) to R13 (a panel), IdoA2S/IdoA(B)(COO-) to R46 (b panel), R47 (c panel) and K114 (d panel) versus MD simulation time in the pentasaccharide (1)-AT (red line) and pentasaccharide (2)-AT (blue line) complexes. Distances are in angstrom (Å), time is in nanoseconds (ns).

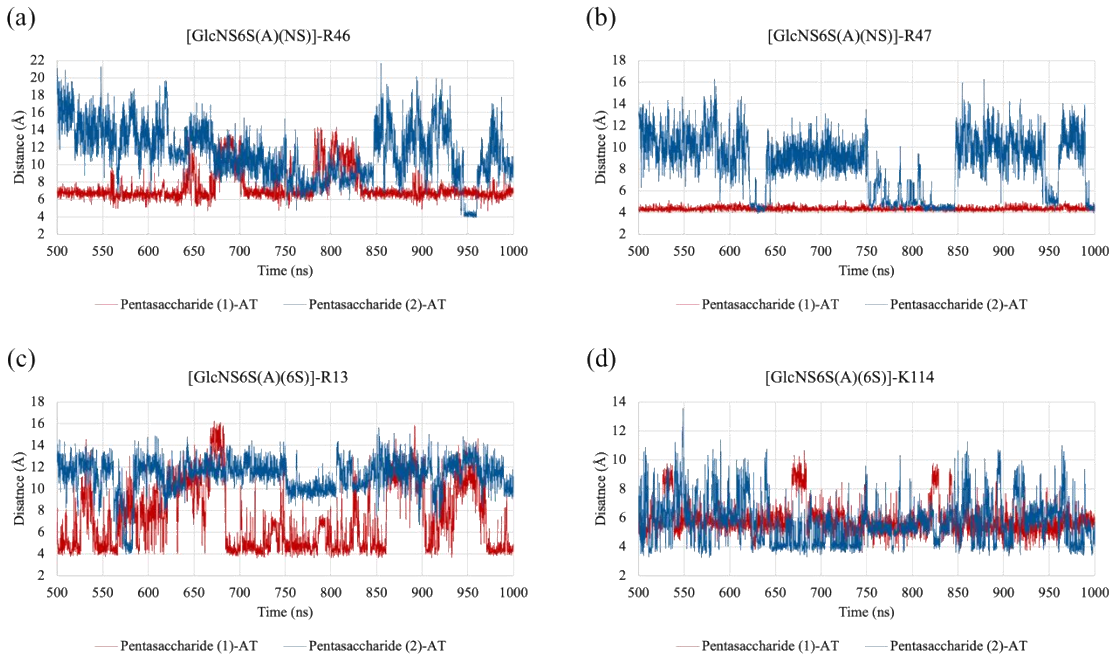

Figure S16. Distances of GlcNS6S(A)(NS) to R46 (a panel) and R47 (b panel), GlcNS6S(A)(6S) to R13 (c panel) and K114 (d panel) versus MD simulation time in the pentasaccharide (1)-AT (red line) and pentasaccharide (2)-AT (blue line) complexes. Distances are in angstrom (Å), time is in nanoseconds (ns).

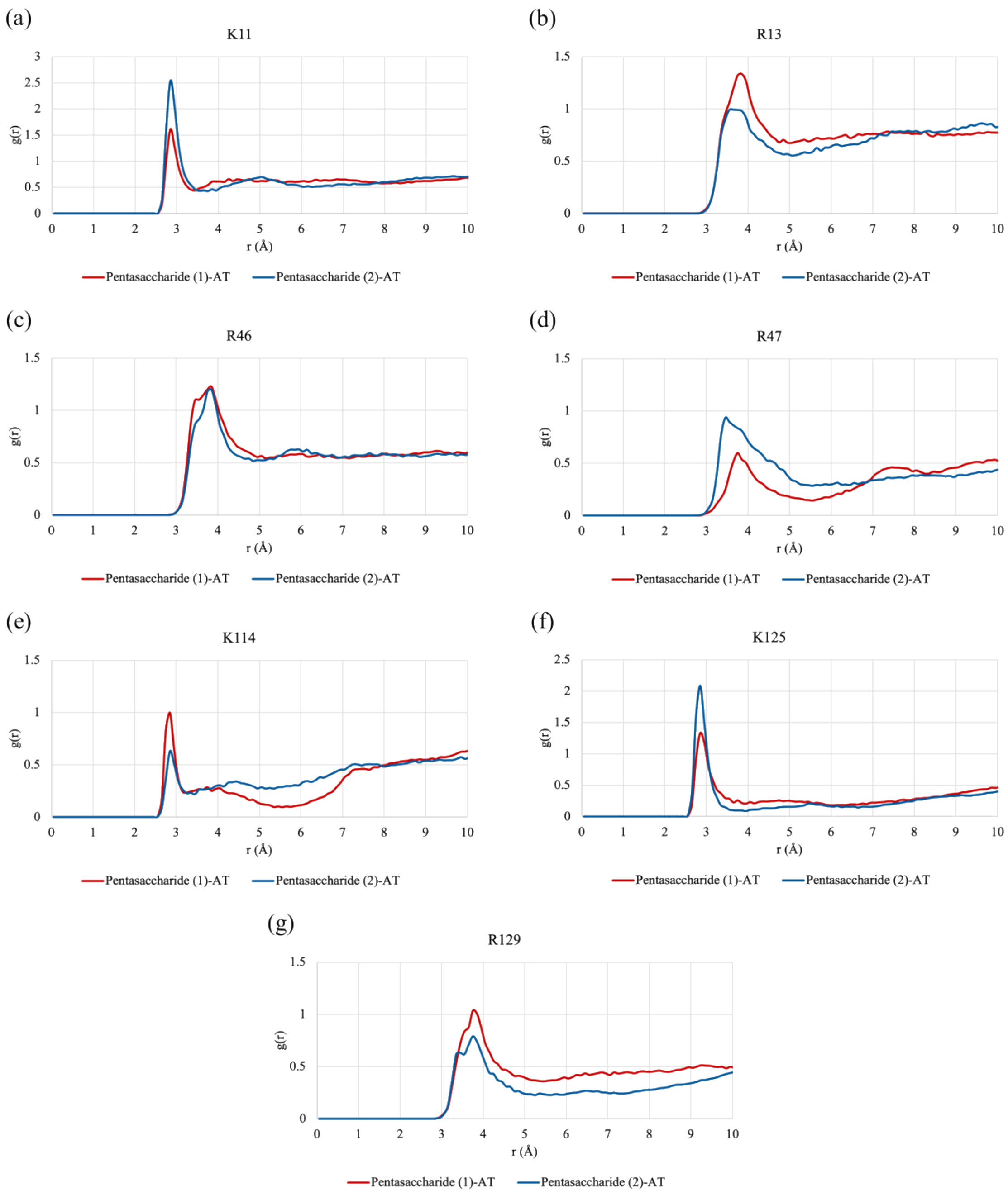

Figure S17. Radial distribution function [g(r)] curves between selected protein residues: K11 (panel a), R13 (b panel), R46 (c panel), R47 (d panel), K114 (e panel), K125 (f panel), R129 (g panel) and water molecules in the pentasaccharide (1)-AT (red line) and pentasaccharide (2)-AT (blue line) complexes. Distances are in angstrom (Å).

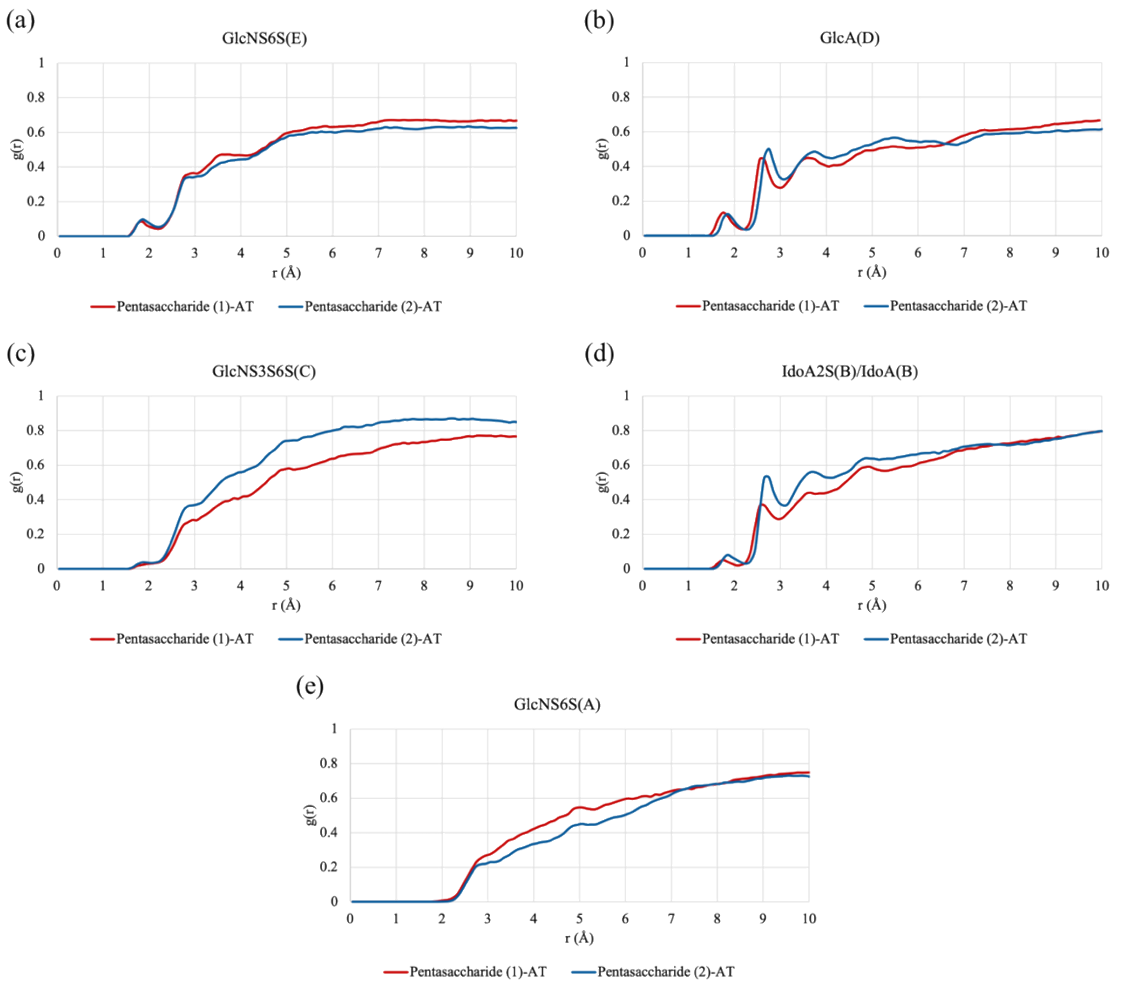

Figure S18. Radial distribution function [g(r)] curves between ligand units: GlcNS6S(E) (a panel), GlcA(D) (b panel), GlcNS3S6S(C) (c panel), IdoA2S/IdoA(B) (d panel), GlcNS6S(A) (e panel) and water molecules in the pentasaccharide (1)-AT (red lines) and pentasaccharide (2)-AT (blue lines) complexes. Distances are in angstrom (Å).

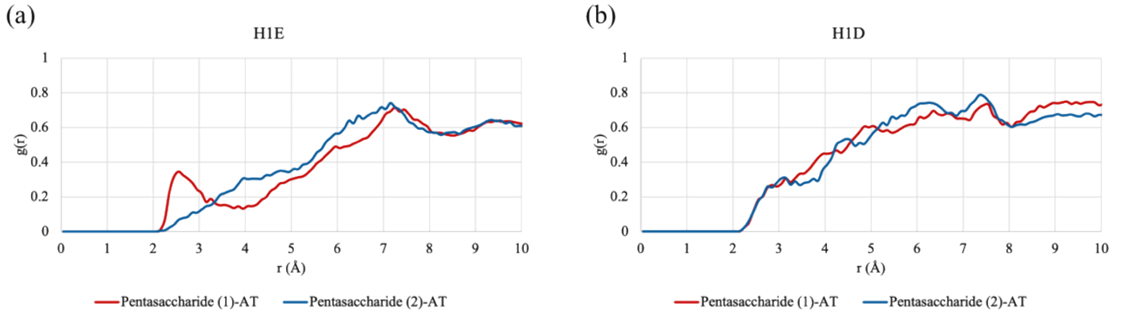

Figure S19. Radial distribution function [g(r)] curves between selected ligand protons: H1 of GlcNS6S(E) (panel a), H1 of GlcA(D) (panel b) and water molecules in the pentasaccharide (1)-AT (red lines) and pentasaccharide (2)-AT (blue lines) complexes. Distances are in angstrom (Å).

Table S12. Comparison between the binding epitopes obtained experimentally (Exp STD) and calculated during MD simulation (Sim STD) of pentasaccharide (1) in complex with AT. The R-factor is < 0.3. The last column reports the average of the simulated STD values (Ave Sim STD).

		500-550 ns	550-600 ns	600-650 ns	650-700 ns	700-750 ns	750-800 ns	800-850 ns	850-900 ns	900-950 ns	950-1000 ns	
**Proton**	**Exp STD**	**Sim STD**	**Sim STD**	**Sim STD**	**Sim STD**	**Sim STD**	**Sim STD**	**Sim STD**	**Sim STD**	**Sim STD**	**Sim STD**	**Ave Sim STD**
H1E	70	100	96	97	96	98	97	100	100	100	100	98
H2E	78	87	85	87	87	86	90	91	88	88	86	88
H5E	69	100	100	100	100	100	100	100	98	99	98	99
H1D	72	55	56	55	54	55	62	58	56	57	62	57
H1C	70	80	76	73	75	76	81	73	74	77	80	76
H2C	100	87	81	84	84	83	89	86	86	92	87	86
H6′C	82	81	78	80	79	76	84	83	86	83	89	82
H6′′C	82	88	89	88	88	86	90	90	89	89	89	89
H1B	64	87	92	89	94	89	91	88	86	87	82	89
H2B	81	89	93	90	94	89	92	88	87	87	85	89
H1A	66	62	65	63	64	62	64	65	68	69	68	65
H2A	95	79	75	73	75	75	73	76	80	79	82	77
H3A	88	73	72	71	71	67	68	70	72	72	74	71
OCH_3(A)	82	55	56	57	55	54	57	55	55	54	62	56
OCH_3(A)	82	52	54	55	53	50	53	54	53	54	59	54
OCH_3(A)	82	50	52	54	51	49	52	51	51	51	61	52

Table S13. Comparison between the binding epitopes obtained experimentally (Exp STD) and calculated during MD simulation (Sim STD) of pentasaccharide (2) in complex with AT. The R-factor is < 0.3. The last column reports the average of the simulated STD values (Ave Sim STD).

		500-550 ns	550-600 ns	600-650 ns	650-700 ns	700-750 ns	750-800 ns	800-850 ns	850-900 ns	900-950 ns	950-1000 ns	
**Proton**	**Exp STD**	**Sim STD**	**Sim STD**	**Sim STD**	**Sim STD**	**Sim STD**	**Sim STD**	**Sim STD**	**Sim STD**	**Sim STD**	**Sim STD**	**Ave Sim STD**
H1E	76	100	100	96	100	100	100	100	100	100	100	97
H2E	81	93	94	100	94	97	99	95	97	98	98	95
H5E	73	85	90	89	88	91	86	87	87	88	92	87
H1D	72	47	49	47	48	53	49	48	46	47	47	50
H1C	65	91	97	99	92	96	94	94	90	91	96	92
H2C	93	62	63	65	63	61	60	57	58	57	61	64
H6′C	66	52	53	55	53	53	51	52	50	51	51	53
H6′′C	66	54	55	61	54	52	54	49	50	48	53	54
H4B	55	45	45	43	42	45	45	42	41	40	42	44
H2A	100	71	76	76	75	76	75	72	69	71	73	76
OCH_3(A)	59	68	72	74	71	72	74	71	68	68	70	70
OCH_3(A)	59	62	65	69	67	67	66	62	63	63	66	65
OCH_3(A)	59	58	65	63	64	64	63	64	60	61	62	62

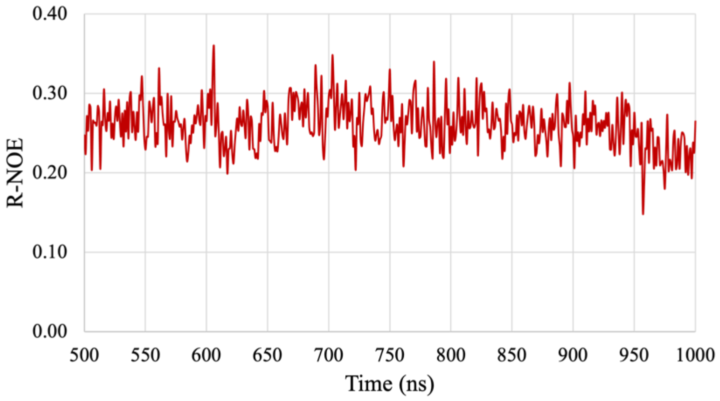

Figure S20. Evolution of the R-factor (R-NOE) of pentasaccharide (1) over the last 500 ns of MD simulation.

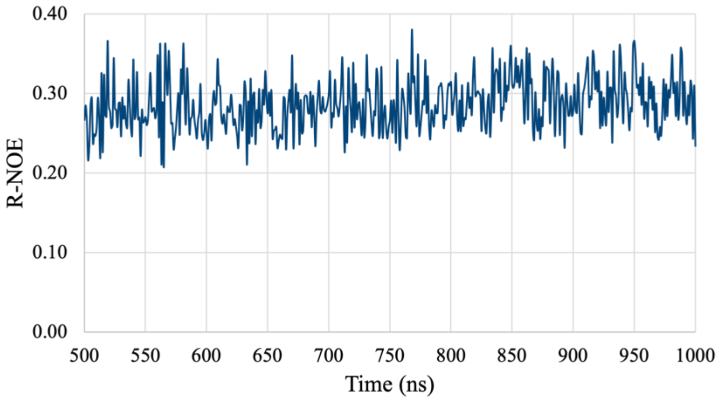

Figure S21. Evolution of the R-factor (R-NOE) of pentasaccharide (2) over the last 500 ns of MD simulation.

Table S14. $^{1}H/^{13}C$ chemical shift assignment of pentasaccharide (1).

Proton	GlcNS6S(E)	GlcA(D)	GlcNS6S(C)	IdoA2S(B)	GlcNS6S(A)
1	5.64/100.2	4.63/103.8	5.53/98.8	5.20/102.2	5.03/101.0
2	3.26/60.7	3.43/75.4	3.46/59.3	4.31/79.7	3.29/60.5
3	3.61/73.8	3.85/78.8	4.37/78.9	4.18/72.6	3.66/72.5
4	3.59/71.7	3.85/79.5	3.98/75.6	4.15/78.6	3.85/79.5
5	3.90/72.4	3.79/78.8	4.16/72.3	4.77/72.8	3.97/71.3
6	4.35-4.43/ 69.4	-	4.27-4.51/ 68.7	-	4.16-4.38/ 69.0
OCH_3	-	-	-	-	3.43/58.1

Table S15. $^{1}H/^{13}C$ chemical shift assignment of pentasaccharide (2).

Proton	GlcNS6S(E)	GlcA(D)	GlcNS6S(C)	IdoA(B)	GlcNS6S(A)
1	5.64/100.3	4.63/104.0	5.39/98.2	5.04/104.9	5.04/101.1
2	3.26/60.7	3.42/75.4	3.46/59.3	3.75/71.4	3.29/60.6
3	3.67/72.7	3.85/78.8	4.39/78.7	4.17/79.5	3.67/72.7
4	3.59/71.7	3.85/79.5	3.99/75.5	4.11/77.3	3.78/79.8
5	4.00/71.2	3.75/81.0	4.07/72.2	4.81/71.0	4.00/71.2
6	4.16-4.38/ 69.0	-	4.22-4.53/ 68.5	-	4.32/69.3
OCH_3	-	-	-	-	3.45/58.1

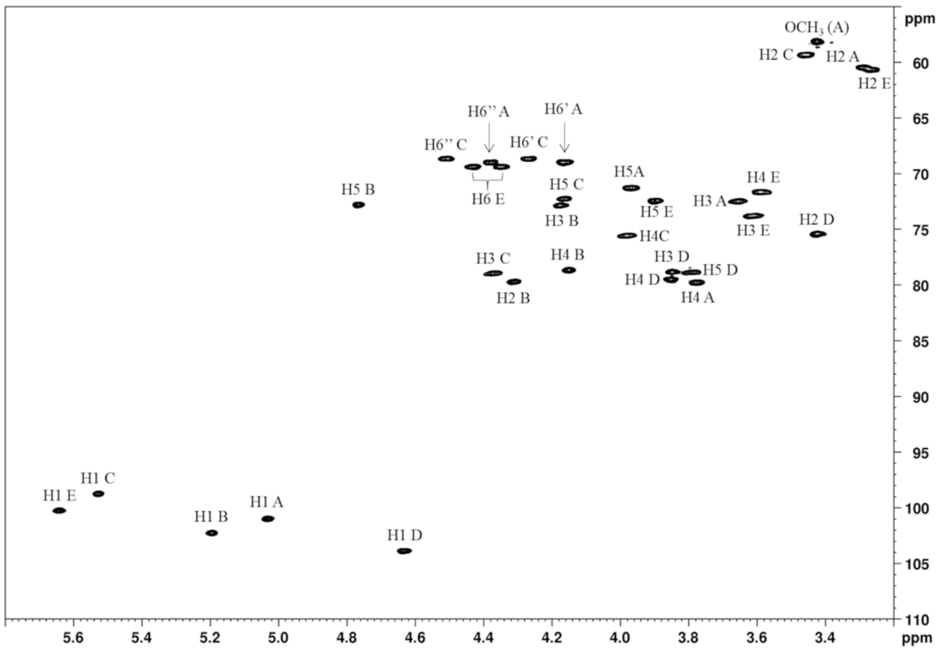

Figure S22. ^{1}H-^{13}C HSQC NMR spectrum of pentasaccharide (1).

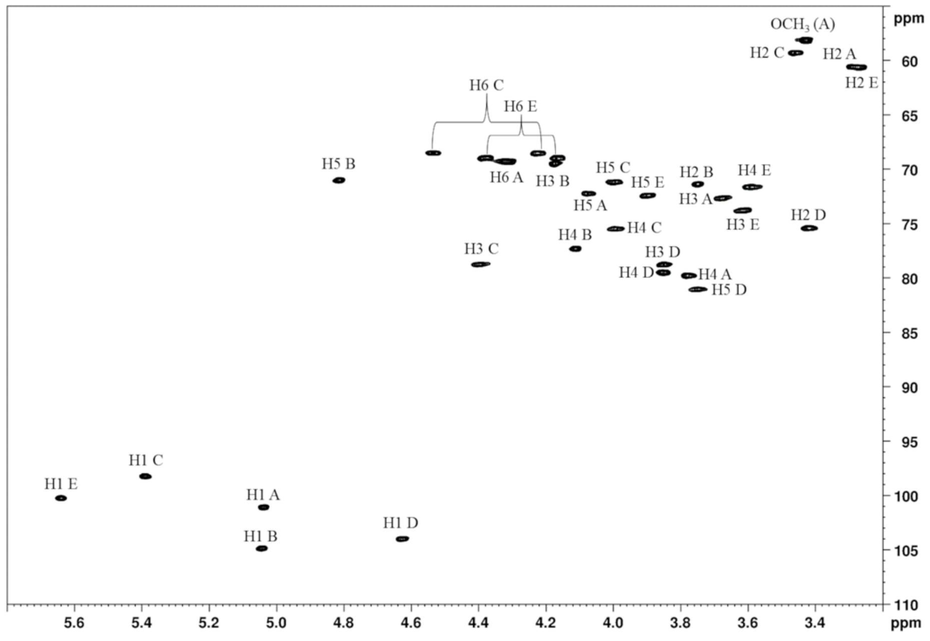

Figure S23. ^{1}H-^{13}C HSQC NMR spectrum of pentasaccharide (2).

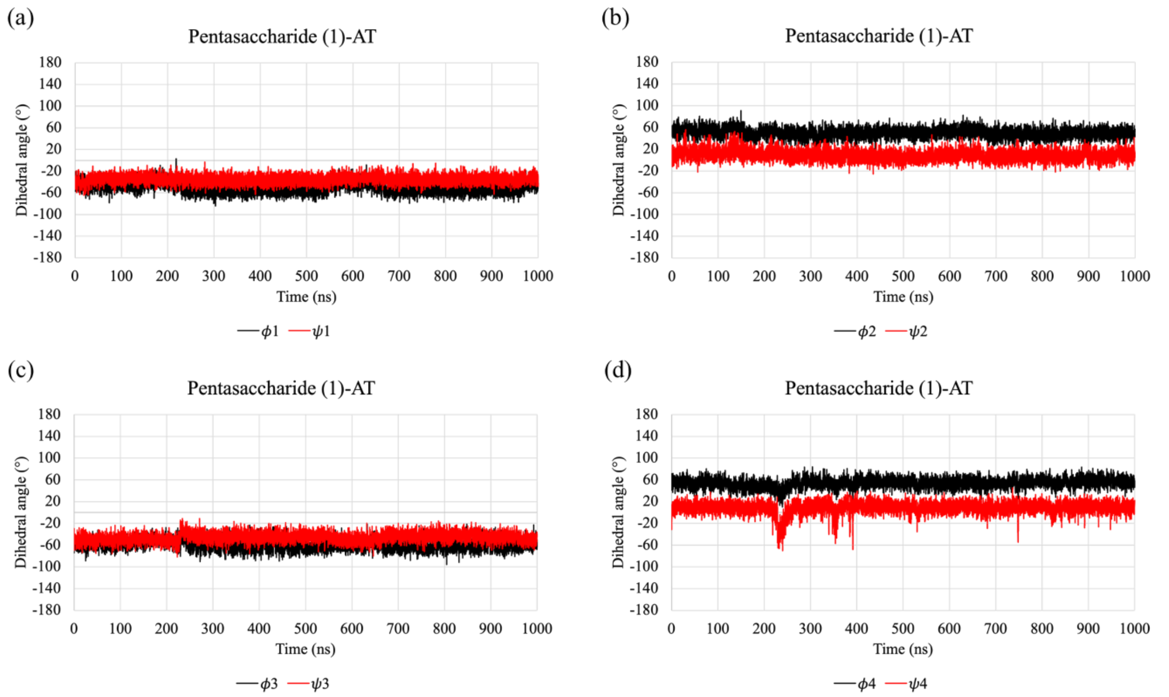

Figure S24. Conformations of the φ_i/ψ_i dihedral angles of pentasaccharide (1) in complex with AT. The pair of dihedral angles: φ_1/ψ_1, φ_2/ψ_2, φ_3/ψ_3, and φ_4/ψ_4 are reported in panels (a), (b), (c), and (d), respectively. Dihedral angles φ_i and ψ_i are represented in black and red lines, respectively

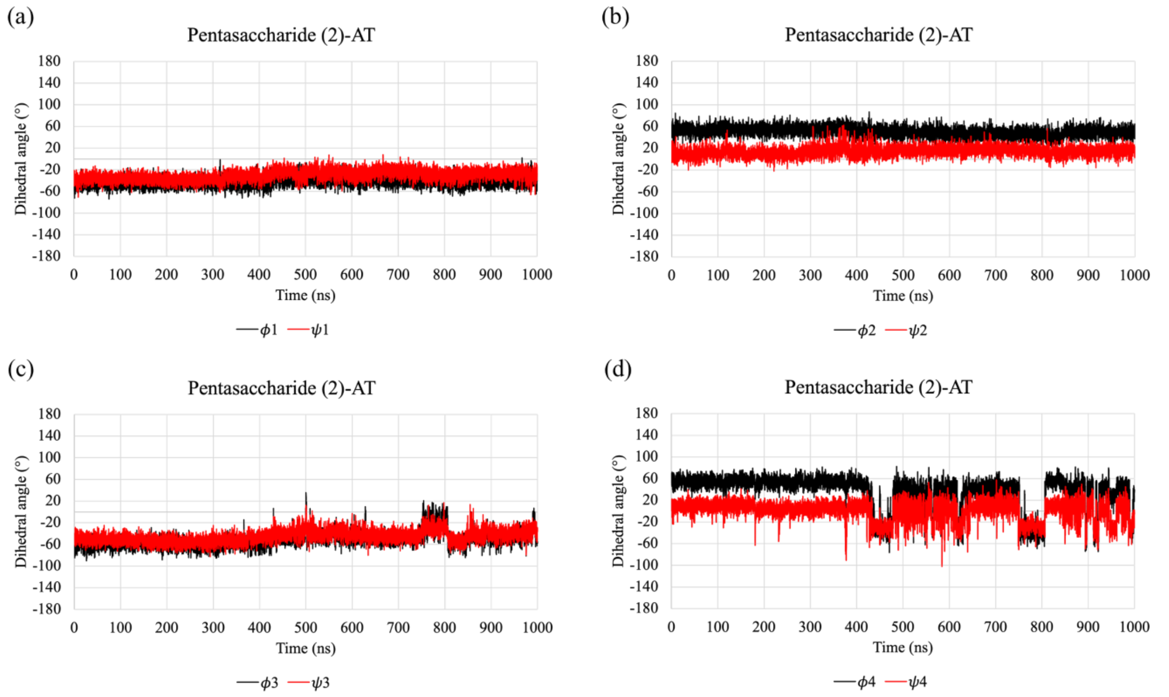

Figure S25. Conformations of the φ_i/ψ_i dihedral angles of pentasaccharide (2) in complex with AT. The pair of dihedral angles: φ_1/ψ_1, φ_2/ψ_2, φ_3/ψ_3, and φ_4/ψ_4 are reported in panels (a), (b), (c), and (d), respectively. Dihedral angles φ_i and ψ_i are represented in black and red lines, respectively
